# Respiratory reoxidation of NADH is a key contributor to high oxygen requirements of oxygen-limited cultures of *Ogataea parapolymorpha*

**DOI:** 10.1093/femsyr/foac007

**Published:** 2022-02-07

**Authors:** Wijbrand J C Dekker, Hannes Jürgens, Raúl A Ortiz-Merino, Christiaan Mooiman, Remon van den Berg, Astrid Kaljouw, Robert Mans, Jack T Pronk

**Affiliations:** Department of Biotechnology, Delft University of Technology, van der Maasweg 9, 2629 HZ Delft, The Netherlands; Department of Biotechnology, Delft University of Technology, van der Maasweg 9, 2629 HZ Delft, The Netherlands; Department of Biotechnology, Delft University of Technology, van der Maasweg 9, 2629 HZ Delft, The Netherlands; Department of Biotechnology, Delft University of Technology, van der Maasweg 9, 2629 HZ Delft, The Netherlands; Department of Biotechnology, Delft University of Technology, van der Maasweg 9, 2629 HZ Delft, The Netherlands; Department of Biotechnology, Delft University of Technology, van der Maasweg 9, 2629 HZ Delft, The Netherlands; Department of Biotechnology, Delft University of Technology, van der Maasweg 9, 2629 HZ Delft, The Netherlands; Department of Biotechnology, Delft University of Technology, van der Maasweg 9, 2629 HZ Delft, The Netherlands

**Keywords:** *Ogataea parapolymorpha*, genome sequence, anaerobic growth, glycerol metabolism, Custers effect, thermotolerance

## Abstract

While thermotolerance is an attractive trait for yeasts used in industrial ethanol production, oxygen requirements of known thermotolerant species are incompatible with process requirements. Analysis of oxygen-sufficient and oxygen-limited chemostat cultures of the facultatively fermentative, thermotolerant species *Ogataea parapolymorpha* showed its minimum oxygen requirements to be an order of magnitude larger than those reported for the thermotolerant yeast *Kluyveromyces marxianus*. High oxygen requirements of *O. parapolymorpha* coincided with a near absence of glycerol, a key NADH/NAD^+^ redox-cofactor-balancing product in many other yeasts, in oxygen-limited cultures. Genome analysis indicated absence of orthologs of the *Saccharomyces cerevisiae* glycerol-3-phosphate-phosphatase genes *GPP1* and *GPP2*. Co-feeding of acetoin, whose conversion to 2,3-butanediol enables reoxidation of cytosolic NADH, supported a 2.5-fold increase of the biomass concentration in oxygen-limited cultures. An *O. parapolymorpha* strain in which key genes involved in mitochondrial reoxidation of NADH were inactivated did produce glycerol, but transcriptome analysis did not reveal a clear candidate for a responsible phosphatase. Expression of *S. cerevisiae GPD2*, which encodes NAD^+^-dependent glycerol-3-phosphate dehydrogenase, and *GPP1* supported increased glycerol production by oxygen-limited chemostat cultures of *O. parapolymorpha*. These results identify dependence on respiration for NADH reoxidation as a key contributor to unexpectedly high oxygen requirements of *O. parapolymorpha*.

## Teaser

Large oxygen requirements of the industrially relevant yeast *O. parapolymorpha* in oxygen-limited cultures were attributed to a necessity to reoxidize surplus NADH from biosynthetic reactions by mitochondrial respiration (‘Custers effect’).

## Introduction

Microbial biotechnology offers promising options for replacing petrochemically produced chemicals with sustainable bio-based alternatives (Weusthuis *et al*. 2011, Thorwall *et al*. 2020). Microbial production of ethanol as a transport fuel, based on plant carbohydrates as renewable feedstocks, is already applied on a large scale. The 87 Mtonnes of ethanol produced worldwide in 2020 (Annual World Fuel Ethanol Production 2020) were almost exclusively produced with the yeast *Saccharomyces cerevisiae* (Jansen *et al*. 2017, Favaro *et al*. 2019). In well-established ‘first-generation’ ethanol processes, this yeast ferments sugars, predominantly derived from corn starch or sugar cane, to ethanol with high productivity, titers, and yields (Basso *et al*. 2011). Use of genetically engineered pentose-fermenting *S. cerevisiae* strains for conversion of lignocellulosic hydrolysates, generated from agricultural residues such as corn stover and sugar cane bagasse, is currently being explored at industrial scale (Jansen *et al*. 2017).

Economic viability of yeast-based ethanol production requires low processing costs and near-theoretical product yields on carbohydrate feedstocks, which are only possible in the absence of respiration (Jansen *et al*. 2017, Favaro *et al*. 2019). Industrial ethanol fermentation is performed in large tanks that readily become and remain anoxic due to vigorous carbon-dioxide production by fermenting yeast cells. The popularity of *S. cerevisiae* for application in these processes is related to its high fermentation rates, innate ethanol tolerance, tolerance to low pH, ability to grow and ferment in the absence of oxygen, and amenability to modern genome-editing techniques (Thomas and Ingledew 1992, van Maris *et al*. 2006, Della-Bianca *et al*. 2013, Lopes *et al*. 2016).

*Saccharomyces cerevisiae* grows optimally at approximately 35°C (Laman Trip and Youk 2020). Fermentation at higher temperatures is industrially attractive as it could reduce cooling costs and, potentially, enable a higher productivity. Additional benefits of thermotolerance may be gained by integrating enzyme-catalyzed polysaccharide hydrolysis and fermentation of the released mono- and di-saccharides in a single unit operation (simultaneous saccharification and fermentation, SSF; Althuri *et al*. 2018). In addition to simplifying industrial processing, SSF could prevent inhibition of hydrolytic enzymes by released monosaccharides (Costa *et al*. 2014, Althuri *et al*. 2018). Moreover, use of thermotolerant yeasts in high-temperature SSF processes can enable a reduction of the required dose of fungal hydrolases, thereby further improving process economy. This advantage is especially relevant for second-generation bioethanol production, in which physically and/or chemically pretreated lignocellulosic plant biomass is hydrolyzed to monomeric sugars by hydrolases with high temperature optima (typically 50–80°C; Alvira *et al*. 2010, Shirkavand *et al*. 2016).

Since supra-optimal temperatures potentially affect all proteins in a cell (Cruz *et al*. 2012), substantial extension of the temperature range of *S. cerevisiae* by metabolic engineering may prove to be an elusive target. Indeed, elegant adaptive laboratory evolution and metabolic engineering studies aimed at improving thermotolerance of *S. cerevisiae*, e.g. by engineering its sterol composition, enabled only modest improvements of its maximum growth temperature (Caspeta *et al*. 2014, Caspeta and Nielsen 2015, Li *et al*. 2021). Use of naturally thermotolerant, facultatively fermentative yeasts such as *Ogataea* sp. (*Hansenula* sp.; Kurtzman 2011) and *Kluyveromyces marxianus*, with temperature maxima of up to 50°C (Hong *et al*. 2007, Kurylenko *et al*. 2014), appears to offer an attractive alternative. Like the large majority of yeast species, these yeasts readily ferment sugars under oxygen-limited conditions (Visser *et al*. 1990, Merico *et al*. 2007, 2009). However, like many other Saccharomycotina yeasts whose evolutionary history did not involve the whole-genome duplication (WGD) event that shaped the genomes of *S. cerevisiae* and closely related species (Wolfe and Shields 1997), *Ogataea parapolymorpha* and *K. marxianus*, cannot grow in the complete absence of oxygen (Visser *et al*. 1990, Blomqvist *et al*. 2012).

Fast anaerobic growth of *S. cerevisiae* requires supplementation of anaerobic growth media with sources of sterols and unsaturated fatty acids (UFAs; Andreasen and Stier 1953, 1954). Omission of UFAs from growth media leads to a drastically reduced growth rate (Dekker *et al*. 2019), which reflects involvement of the oxygen-consuming cytochrome-b5 Δ9-desaturase Ole1 (Stukey *et al*. 1989) in UFA synthesis. Similarly, a strict sterol requirement of anaerobic *S. cerevisiae* cultures reflects involvement of multiple mono-oxygenases in sterol biosynthesis (Henneberry and Sturley 2005). Because of these biosynthetic oxygen requirements, media for anaerobic cultivation of *S. cerevisiae* are routinely supplemented with ergosterol and Tween 80, an oleate ester that serves as UFA source (Andreasen and Stier 1954). Additional oxygen requirements of *S. cerevisiae* for synthesis of biotin, nicotinate, pantothenate, and thiamine (Wightman and Meacock 2003, Perli *et al*. 2020, Wronska *et al*. 2021) generally go unnoticed in laboratory studies due to their routine inclusion in synthetic media (Perli *et al*. 2020). In most non-*Saccharomyces* yeasts, pyrimidine synthesis imposes an additional biosynthetic oxygen requirement, as it depends on a mitochondrial, respiration-coupled Class-II dihydroorotate dehydrogenase (DHODase, Ura9). In contrast, *S. cerevisiae* only harbors a cytosolic, respiration-independent Class-I-A DHODase (Ura1), which uses fumarate as electron acceptor (Nagy *et al*. 1992, Wolfe and Shields 1997, Langkjær *et al*. 2003, Riley *et al*. 2016). *Kluyveromyces* sp. contain both Ura1 and Ura9 orthologs, whose expression is regulated in response to oxygen availability (Dekker *et al*. 2021).

Development of metabolic engineering strategies for eliminating oxygen requirements of non-*Saccharomyces* yeasts requires elucidation of underlying oxygen- and/or respiration-dependent biochemical reactions. To investigate oxygen requirements of the thermotolerant yeast *O. parapolymorpha*, its physiological and transcriptional responses to oxygen limitation were studied in chemostat cultures and compared to recent literature data on *K. marxianus* and *S. cerevisiae* (Dekker *et al*. 2021). Based on the results of these experiments, co-feeding of acetoin was used to explore the impact of cytosolic NADH oxidation on the physiology of *O. parapolymorpha* in oxygen-limited cultures. In additional the genome of *O. parapolymorpha* was searched for orthologs of genes implicated in the (in)ability of other yeasts to grow anaerobically and, in particular, in the production of glycerol as ‘redox sink’ for reoxidation of NADH formed in biosynthetic reactions. Glycerol metabolism in *O. parapolymorpha* was further investigated in a mutant strain in which key genes involved in mitochondrial, respiration-linked NADH oxidation were deleted. Metabolic engineering of redox metabolism in *O. parapolymorpha* was explored by expressing *S. cerevisiae* genes involved in glycerol production.

## Methods

### Strain maintenance

*Ogataea parapolymorpha* CBS11895 (DL-1) was obtained from the Westerdijk Fungal Biodiversity Institute (Utrecht, The Netherlands). For propagation and maintenance, cultures were grown on yeast extract–peptone–dextrose (YPD) medium (10 g l^–1^ Bacto yeast extract, 20 g l^–1^ Bacto peptone, and 7.5 g l^–1^ glucose) in an Innova shaker incubator (New Brunswick Scientific, Edison, NJ) set at 30°C and 200 rpm. YPD was prepared by autoclaving (20 min at 121°C) a solution of yeast extract and peptone and then aseptically adding a separately autoclaved (20 min at 110°C) concentrated glucose solution. Fro−en stock cultures, prepared from exponentially growing cultures by addition of glycerol to a final concentration of 30% (v/v), were aseptically stored at −80°C.

### Molecular biology techniques

PCR amplification for cloning was performed with Phusion High Fidelity polymerase (Thermo Fisher Scientific, Waltham, MA) according to the manufacturer's instructions. DreamTaq polymerase (Thermo Fisher Scientific) was used for diagnostic PCR with yeast genomic DNA, isolated with the LiAc/SDS method (Lõoke *et al*. 2011) from overnight cultures on YPD, as template. Desalted or PAGE-purified oligonucleotide primers (Sigma-Aldrich, St. Louis, MO) are listed in Table S1 (Supporting Information). PCR-amplified DNA fragments were analyzed by gel electrophoresis and, when required, purified from agarose gels with a Zymoclean Gel DNA Recovery Kit (Zymo Research, Irvine, CA). Prior to purification, template plasmid DNA was removed by FastDigest *DpnI* digestion (Thermo Fisher Scientific). Alternatively, DNA fragments were purified with a GenElute PCR Clean-Up Kit (Sigma-Aldrich). Gibson assembly with the NEBuilder HiFi DNA Assembly Master mix (New England Biolabs, Ipswich, MA), was performed with a down-scaled reaction volume of 5 µl and a total incubation time at 50°C of 1 h. The GenElute Plasmid Miniprep kit (Sigma-Aldrich) was used for plasmid isolation from overnight cultures of *Escherichia coli* XL1-Blue, which was used for plasmid amplification and storage.

### Plasmid construction

Plasmids used in this study are described in Table 1. Promoter and terminator sequences of Op*PMA1* and Op*TEF1*, which encode plasma-membrane H^+^-ATPase and translation elongation factor EF-1α, respectively, were chosen based on high transcript levels across a range of specific growth rates (Juergens *et al*. 2020). Promoter and terminator fragments were defined as regions 800 bp upstream and 300 bp downstream, respectively, of coding sequences. For targeted integration into a genetic locus, 500 bp flanking homology regions were designed to partially delete the target region, without altering promoter (800 bp) or terminator (300 bp) sequences of adjacent genes.

**Table 1.** Plasmids used in this study. Superscripts indicate restriction sites and DNA sequences for homologous recombination are indicated with (HR). Sc: *Saccharomyces cerevisiae*, Op: *Ogataea parapolymorpha*, Ag: *Ashbya gossypii*, Aa: *Arxula adeninivorans*, and Sp: *Streptococcus pyogenes*.

Plasmid	Characteristics	Source
pUC19	ori ampR	Norrander *et al*. (1983)
pUD803	NDH2-3(HR) Aa*TEF1*p-nat1-Sc*PHO5*t *NDH2-3*(HR)	Juergens *et al*. (2021)
pUD1069	ori amp^R^*gbu1*(HR) Op*PMA1*p-Sc*GPP1*-Op*PMA1*t-Ag*TEF1*p-nat1-Sc*ADH1*t *gbu1*(HR)	This study
pUD1082	ori ampR *sga1*(HR) Op*TEF1*p-Sc*GPD2*-Op*TEF1*t Ag*TEF1*p-hph-Ag*TEF1*t *sga1*(HR)	This study
pUDP002	panARS Ag*TEF1*p-hph-Ag*TEF1*t Sc*TDH3*p^BsaI BsaI^Sc*CYC1*t Aa*TEF1*p-Spcas9-Sc*PHO5*t	Juergens *et al*. (2018b)
pUG6	ori ampR loxP-kanMX-loxP	Güldener *et al*. (1996)

Plasmid pUD1069 (Sc*GPP1*) was constructed by Gibson assembly of fragments amplified with 20-bp terminal overlapping extensions. The Sc*GPP1* coding sequence was PCR amplified from genomic DNA of *S. cerevisiae* CEN.PK113-7D with primer pair 15183/15184. Promoter and terminator fragments of Op*PMA1* were amplified from genomic DNA of strain CBS11895 with primer pairs 15185/15186 and 15191/15193, respectively. Up- and down-stream 500-bp homology flanks to the Op*GBU1* locus (Romagnoli *et al*. 2014) were amplified with primer pairs 15192/15196 and 15195/15198, respectively. The natNT2 marker (Ag*TEF1*p-nat1-Sc*ADH1*t) from *Streptomyces noursei* (Krügel *et al*. 1993, Goldstein and McCusker 1999, Janke *et al*. 2004) was amplified from pUD803 (Juergens *et al*. 2021) with primer pair 15187/15188. The pUG6 backbone was PCR amplified with primer pair 15189/15190. Mixing and Gibson assembly of equimolar amounts of the resulting fragments yielded plasmid pUD1069, whose correct assembly was verified by restriction analysis and diagnostic PCR with primers 15224, 15225, 15226, 15227, 15228, 15229, 15230, 15231, and 15232.

To construct an Sc*GPD2* expression cassette, its coding sequence was PCR amplified from genomic DNA of CEN.PK113-7D with primer pair 15745/15744. Op*TEF1* promoter and terminator fragments were PCR-amplified from genomic DNA with primer pairs 15749/15748 and 15746/15747, respectively. Upstream and downstream 800-bp recombination flanks for integration at the Op*SGA1* integration locus were PCR amplified with primer pairs 15740/15739 and 15735/15736, respectively. A *Klebsiella pneumoniae hph* (Hyg^R^) expression cassette, using the Ag*TEF1* promoter and Ag*TEF1* terminator from *Ashbya gossypii*, was PCR amplified from pUDP002 (Juergens *et al*. 2018b) with primer pair 1312/15743. The pUC19 backbone was PCR amplified with primer pair 15738/15737. Mixing and Gibson assembly of equimolar amounts of the resulting six fragments yielded plasmid pUD1082 (Sc*GPD2*), whose correct assembly was verified by restriction analysis.

### Strain construction

Yeast strains used in this study are described in Table 2. *Ogataea parapolymorpha* strains were transformed by electroporation of freshly prepared electrocompetent cells (Juergens *et al*. 2018b). Transformants were selected on YPD agar containing hygromycin B (300 µg ml ^–1^) or nourseothricin (100 µg ml ^–1^). Strains IMX2119, IMX2587, and IMX2588 were constructed with the split-marker integration approach (Fairhead *et al*. 1996), with approximately 480-bp overlapping homology sequences for marker recombination and genome integration. The natNT2 split-marker fragments for integration of an Sc*GPP1* expression cassette into the Op*GBU1* locus were amplified from pUD1069 (Sc*GPP1*) with primer pairs 15192/15194 and 15196/15197, yielding two integration fragments with a homologous sequence overlap. Similarly, *hph* split-marker fragments for integration of an Sc*GPD2* cassette were constructed by amplification from pUD1082 (Sc*GPD2*) with primer pairs 15740/15741 and 15742/15736. Correct integration of the split-marker fragments at the Op*GBU1* locus was verified by diagnostic PCR with primers 15192, 15197, 15233, and 15234 and integration at the Op*SGA1* locus with primers 15894, 15748, and 15895.

**Table 2.** Yeast strains used in this study. Sc: *Saccharomyces cerevisiae* and Op: *Ogataea parapolymorpha*.

Genus	Strain	Relevant genotype	Reference
*S. cerevisiae*	CEN.PK113-7D	*MATa URA3 HIS3 LEU2 TRP1 MAL2-8c SUC2*	Entian and Kötter (2007)
*O. parapolymorpha*	CBS11895	-	Westerdijk
*O. parapolymorpha*	IMX2119	*gbu1Δ*::Op*PMA1*p-Sc*GPP1*-Op*PMA1*t natNT2	This study
*O. parapolymorpha*	IMX2587	*sga1Δ::*Op*TEF1*p-Sc*GPD2*-Op*TEF1*t-hph	This study
*O. parapolymorpha*	IMX2588	Op*gbu1Δ*::Op*PMA1*p-Sc*GPP1*-Op*PMA1*t natNT2 Op*sga1Δ::*Op*TEF1*p-Sc*GPD2*-Op*TEF1*t-hph	This study
*O. parapolymorpha*	IMX2167	Op*ndh2-1Δ::kanR* Op*ndh2-2Δ::hph* Op*ndh2-3::natNT2* Op*gut2Δ::pat*	Juergens *et al*. (2021)

### Bioreactor cultivation

Chemostat cultures of *O. parapolymorpha* strains were grown in 2-l bioreactors (Applikon Biotechnology, Delft, The Netherlands) with a working volume of 1.2 l, operated at a dilution rate of 0.1 h^–1^, at pH 6, at 30°C, and at a stirrer speed of 800 rpm. Oxygen-limited chemostat cultures were sparged at a rate of 0.5 l min^–1^ (0.4 vvm) with a mixture of N_2_ and air that contained 840 ppm O_2_, and aerobic cultures with air (21 × 10^4^ ppm O_2_). Cultures were fed with a synthetic medium with vitamins and with urea as nitrogen source (Luttik *et al*. 2000), supplemented with 7.5 g l^–1^ glucose (aerobic cultures) or 20 g l^–1^ glucose (oxygen-limited cultures) and 0.2 g l^–1^ Pluronic 6100 PE antifoam (BASF, Ludwigshafen, Germany). An 800-fold concentrated solution of the anaerobic growth factors Tween 80 (polyethylene glycol sorbitan monooleate; Merck, Darmstadt, Germany), ergosterol (≥ 95% pure; Sigma-Aldrich) in ethanol was prepared and added to sterile media as described previously (Dekker *et al*. 2019), but with a 5-fold lower Tween 80 concentration. Concentrations of Tween 80, ergosterol and ethanol in reservoir media of oxygen-limited cultures were 84 mg l^–1^, 10 mg l^–1^, and 0.67 g l^–1^, respectively. Tween 80 was omitted from media for aerobic cultivation to prevent excessive foaming. Where indicated, a filter-sterilized acetoin solution was added to a concentration of 2.0 g l^–1^. Before autoclaving, bioreactors were checked for gas leakage by submersion in water while applying a 0.3 bar overpressure. Bioreactors were equipped with Fluran tubing and Viton O-rings and the glass medium reservoir was equipped with Norprene tubing and continuously sparged with pure nitrogen gas to minimize oxygen entry. Inocula for bioreactor cultures were prepared by harvesting an exponentially growing 100-ml shake-flask culture on synthetic medium with glucose by centrifugation (5 min at 4000 x *g*) and washing the biomass once with sterile demineralized water. Oxygen-limited chemostat cultures were started from aerobic bioreactor batch cultures on synthetic medium containing 1.5 g l^–1^ glucose. When CO_2_ production in these batch cultures had reached a maximum and started to decline, chemostat cultivation was initiated by applying a constant medium feed rate and continuous effluent removal. Chemostat cultures were assumed to have entered steady state when, at least 5 volume changes after a change in growth conditions, the biomass concentration and specific carbon dioxide production rate differed by less than 10% over three samples separated by at least one volume change.

Aerobic bioreactor batch and chemostat (D = 0.1 h^–1^) cultures of *O. parapolymorpha* CBS11895 and IMX2167 (Table 5) were grown at 30°C and at pH 5 in 2-l bioreactors (Applikon Biotechnology) with a working volume of 1.0 l, on a synthetic medium (Verduyn *et al*. 1990) containing 7.5 g l^–1^ glucose and 20 g l^–1^ glucose, respectively, and ammonium sulfate as nitrogen source.

### Analytical methods

Off-gas analysis, biomass dry weight measurements, optical density measurements, metabolite HPLC analysis of culture supernatants, and correction for ethanol evaporation in bioreactor experiments were performed as described previously (Dekker *et al*. 2021). Rates of substrate consumption and metabolite production were calculated from glucose and metabolite concentrations in steady-state cultures, analyzed after rapid quenching of culture samples (Mashego *et al*. 2003). Recoveries of carbon and degree of reduction (Roels 1980) were calculated based on concentrations of relevant components in medium feed, culture samples, and in- and out-going gas streams. For organic compounds that only contain carbon, hydrogen, and/or oxygen, degree of reduction (*γ*) represents the number of electrons released upon complete oxidation to CO_2_, H_2_O and or H^+^. These oxidized compounds are assigned a *γ* of zero, which yields defined values of *γ* for H, C, and O of 1, 4, and 2, respectively and for positive and negative charge of −1 and 1, respectively. To simplify construction of degree-of-reduction balances, the nitrogen source is generally assigned *γ* = 0 which, with NH_4_^+^ as nitrogen source, implies that *γ* = 3 for N. Calculations were based on an estimated degree of reduction and carbon content of yeast biomass (Lange and Heijnen 2001).

### Genome sequencing and assembly

Cells were harvested from an overnight culture on YPD by centrifugation (5 min at 4000 x *g*) and genomic DNA was isolated with the Qiagen genomic DNA 100/G Kit (Qiagen, Hilden, Germany) according to the manufacturer's instructions. MinION genomic DNA libraries (SQK-LSK108, Oxford Nanopore Technologies, Oxford, UK) were prepared using the 1D genomic DNA by ligation and the SQK-LSK108 library was sequenced on an R9 chemistry flow cell (FLO-MIN107). Base calling was performed with Albacore v1.1.5 (Oxford Nanopore Technologies), reads were assembled using Canu v1.4 (Koren *et al*. 2017), and the resulting assembly was polished with Pilon v1.18 (Walker *et al*. 2014). To annotate the genome sequence of *O. parapolymorpha* CBS11895, pooled RNAseq libraries were used to generate a *de novo* transcriptome assembly using Trinity (v2.8.3; Grabherr *et al*. 2011) and then entered into the PASA pipeline (Singh *et al*. 2017) as implemented in funannotate v1.7.7 (Palmer and Stajich 2019). RNA reads from *O. parapolymorpha* CBS11895 batch and chemostat cultures (Juergens *et al*. 2020) were downloaded from NCBI (www.ncbi.nlm.nih.gov) with the Gene Expression Omnibus accession number GSE140480. Funannotate Compare was used to obtain (co)ortholog groups of genes generated with ProteinOrtho5 (Lechner *et al*. 2011). Publicly available genome annotations of *S. cerevisiae* S288C (GCF_000146045.2) and a previous version of *O. parapolymorpha* (DL-1; GCF_000187245.1) were then used to functionally annotate, guided by ortholog assignment, the new CBS11895 genome sequence.

### RNA extraction, isolation, sequencing, and transcriptome analysis

Biomass samples from batch and chemostat cultures were directly sampled into liquid nitrogen to prevent mRNA turnover (Piper *et al*. 2002). Processing of samples for storage at −80°C and RNA isolation were performed as described previously (Dekker *et al*. 2021). Batch cultures were sampled when, in the exponential growth phase, approximately 25% of the initially supplied glucose had been consumed (Juergens *et al*. 2020). Quality of isolated RNA was analyzed with an Agilent Tapestation (Agilent Technologies, CA) using RNA screen tape (Agilent). RNA concentrations were measured with a Qubit RNA BR assay kit (Thermo Fisher Scientific). The TruSeq Stranded mRNA LT protocol (Illumina, San Diego, CA) was used to generate RNA libraries for paired-end sequencing by Macrogen (Macrogen Europe, Amsterdam, The Netherlands) with a read length of 151 bp on a NovaSeq sequencer (Illumina). RNA reads were mapped to the genome of *O. parapolymorpha* CBS11895 (Juergens *et al*. 2020) using bowtie (v1.2.1.1; Langmead *et al*. 2009). Alignments were filtered and sorted using samtools (v1.3.1; Li *et al*. 2009) as described previously (Dekker *et al*. 2021). Reads were counted with featureCounts (v1.6.0; Liao *et al*. 2014) of which both pairs of the paired-end reads were aligned to the same chromosome. EdgeR (v3.28.1; McCarthy *et al*. 2012) was used to perform differential gene expression and genes with fewer than 10 reads per million in all conditions were eliminated from subsequent analysis. Counts were normalized using the trimmed mean of M values (TMM; Robinson and Oshlack 2010) method and the dispersion was estimated using generalized linear models. Differential expression was calculated using a log ratio test adjusted with the Benjamini–Hochberg method. Absolute log 2 fold-change values (> 2), false discovery rate (< 0.5), and *P*-value (< .05) were used as significance cut-offs.

Gene set analysis (GSA) based on gene ontology (GO) terms with Piano (v2.4.0; Väremo *et al*. 2013) was used for functional interpretation of differential gene expression profiles. Interproscan (Jones *et al*. 2014) was used to assign GO terms to the genome annotation of *O. parapolymorpha*. Co-ortholog groups of genes were generated with ProteinOrtho5 (Lechner *et al*. 2011) as implemented in the funannotate pipeline and used to homogenize GO terms for co-ortholog groups as described previously (Dekker *et al*. 2021). GSA was done with Piano (v2.4.0; Väremo *et al*. 2013) and gene statistics were calculated with Stouffer, Wilcoxon rank-sum test and reporter methods as implemented in Piano. Consensus gene level statistics were obtained by *P*-value and rank aggregation and considered significant when absolute log 2 fold-change values > 1. ComplexHeatmap (v2.4.3; Gu *et al*. 2016) was used to visualize differentially expressed genes. To interpret the GO-term based GSA between three yeast species in response to oxygen limitation, hierarchical clustering (complete method and Euclidian distance) in R (R Core Team 2021) was performed on GO-terms from biological process category. Clustering was based on the number of overlapping distinct directionality *P*-values in the three yeast species with a significance *P*-value cut-off of .01.

### Sequence homology searches

*Saccharomyces cerevisiae* protein sequences were used as queries to search whole-genome sequences of 16 *Ogataea* species, *K. marxianus, Candida arabinofermentans*, and *Brettanomyces bruxellensis* with tblastn (blast.ncbi.nlm.nih.gov; Camacho *et al*. 2009). Significance was based on alignment criteria, with an e-value of < 10^–7^, > 70% alignment coverage and > 50% nucleotide identity. Blast results were mapped to a subtree of selected yeast species in the phylum Ascomycota (Shen *et al*. 2020) using Treehouse (Steenwyk and Rokas 2019) to subset the phylogenetic tree.

## Results

### Oxygen requirements of *O. parapolymorpha* in oxygen-limited chemostat cultures

Chemostat cultivation enables analysis of impacts of different process parameters at a fixed specific growth rate, which in ideally mixed, steady-state chemostat cultures equals the dilution rate (D, h^–1^). Oxygen requirements of the wild-type *O. parapolymorpha* strain CBS11895 (DL-1; Suh and Zhou 2010) were quantitatively assessed by comparing its physiology under two aeration regimes in glucose-grown chemostat cultures operated at D = 0.1 h^–1^ (Table 3). Results from this analysis were compared with data that were previously obtained, under the same cultivation conditions, with *S. cerevisiae* CEN.PK113-7D and *K. marxianus* CBS6556 (Dekker *et al*. 2021).

**Table 3.** Physiological parameters of chemostat cultures (D = 0.1 h^–1^, 30°C) of *O. parapolymorpha, K. marxianus*, and *S. cerevisiae*, grown on glucose under aerobic (21 × 10^4^ ppm O_2_ in inlet gas, 7.5 g l^–1^ glucose in feed medium) or oxygen-limited (840 ppm O_2_ in inlet gas; 20 g l^–1^ glucose in feed medium) conditions. Data for *K. marxianus* and *S. cerevisiae* were obtained from a previous study (Dekker *et al*. 2021). Growth media were supplemented with ergosterol and Tween 80, except for media for aerobic cultures of *O. parapolymorpha*, from which Tween 80 was omitted to prevent excessive foaming. Data are represented as mean ± standard deviation of data obtained from replicate chemostat cultures. Negative and positive biomass-specific conversion rates (q) represent consumption and production rates, respectively, with subscript x denoting biomass dry weight. B.D.: below detection limit (concentration < 0.1 mM) and (-): not applicable due to co-consumption of ethanol added as ergosterol solvent.

	*O. parapolymorpha* CBS11895		*K. marxianus* CBS6556		*S. cerevisiae* CEN.PK113-7D	
Yeast strain	Aerobic	O_2_-limited	Aerobic	O_2_-limited	Aerobic	O_2_-limited
O_2_ in inlet gas (ppm)	21 × 10^4^	840	21 × 10^4^	840	21 × 10^4^	840
Replicates	2	2	2	5	3	3
Biomass (g_x_ l^–1^)	4.33 ± 0.06	0.62 ± 0.01	3.79 ± 0.03	1.57 ± 0.22	4.22 ± 0.11	2.29 ± 0.07
Residual glucose (g l^–1^)	B.D.	15.92 ± 0.01	B.D.	0.10 ± 0.03	B.D.	0.07 ± 0.01
**Biomass-specific conversion rates**						
Specific growth rate (h^–1^)	0.10 ± 0.00	0.10 ± 0.01	0.10 ± 0.00	0.11 ± 0.01	0.10 ± 0.00	0.10 ± 0.00
q_glucose_ (mmol g_x_^–1^ h^–1^)	–0.94 ± 0.04	–3.67 ± 0.20	–1.05 ± 0.00	–7.46 ± 0.66	–0.95 ± 0.05	–4.59 ± 0.18
q_ethanol_ (mmol g_x_^–1^ h^–1^)	–0.48 ± 0.08	4.75 ± 0.33	–0.52 ± 0.00	11.49 ± 0.97	–0.44 ± 0.05	7.48 ± 0.17
q_glycerol_ (mmol g_x_^–1^ h^–1^)	B.D.	0.02 ± 0.01	B.D.	1.12 ± 0.12	B.D.	0.45 ± 0.01
q_succinate_ (mmol g_x_^–1^ h^–1^)	B.D.	0.00 ± 0.00	B.D.	0.02 ± 0.01	B.D.	0.00 ± 0.00
q_O2_ (mmol g_x_ h^–1^)	–2.35 ± 0.15	–0.60 ± 001	–3.52 ± 0.07	–0.23 ± 0.05	–2.61 ± 0.20	–0.15 ± 0.01
q_CO2_ (mmol g_x_ h^–1^)	2.89 ± 0.21	6.42 ± 0.30	3.73 ± 0.04	10.5 ± 1.1	2.82 ± 0.17	7.91 ± 0.91
**Stoichiometries**						
RQ (-qCO_2_/qO_2_)	1.23 ± 0.15	10.7 ± 0.3	1.06 ± 0.01	49.3 ± 16.8	1.08 ± 0.04	52.2 ± 4.2
Y_glycerol/X_ (mmol g_x_^–1^)	B.D.	0.24 ± 0.07	B.D.	10.7 ± 1.5	B.D.	4.66 ± 0.13
Y_X/glucose_ (g_x_ g^–1^)	0.59 ± 0.01	0.15 ± 0.02	0.53 ± 0.00	0.08 ± 0.01	0.57 ± 0.01	0.12 ± 0.00
Y_ethanol/glucose_ (g g^–1^)	-	0.33 ± 0.04	-	0.39 ± 0.00	-	0.42 ± 0.03
Y_X/O2_ (g_x_ mmol^–1^)	0.04 ± 0.00	0.17 ± 0.01	0.03 ± 0.01	0.50 ± 0.18	0.04 ± 0.00	0.64 ± 0.06
**Recoveries (out/in)**						
Carbon (%)	100.3 ± 0.6	94.4 ± 6.2	100.5 ± 0.1	91.1 ± 4.5	99.9 ± 1.1	101.2 ± 5.7
Degree of reduction (%)	97.8 ± 0.8	92.0 ± 6.1	98.8 ± 0.2	94.5 ± 1.0	98.4 ± 1.2	100.1 ± 1.4

In fully aerobic chemostat cultures sparged with air (0.5 l min^–1^), growth of *O. parapolymorpha* was glucose limited and sugar dissimilation occurred exclusively *via* respiration, as indicated by a respiratory quotient (RQ) close to 1 (Table 3). The apparent biomass yield on glucose in aerobic cultures was approximately 10% higher than previously reported (Verduyn *et al*. 1991) due to co-consumption of ethanol, which was used as solvent for ergosterol. When cultures were instead sparged with a mixture of N_2_ and air (0.5 l min^–1^, oxygen content 840 ppm), the apparent biomass yield on glucose in steady-state cultures was 4-fold lower than in the aerobic cultures (0.15 g g^–1^ and 0.59 g g^–1^, respectively, Table 3). A high residual glucose concentration (15.9 g l^–1^) indicated that growth in these cultures was limited by oxygen rather than by glucose. Respiro-fermentative glucose dissimilation by the oxygen-limited cultures was evident from an RQ of 10.7 and a specific ethanol-production rate of 4.8 mmol (g biomass)^–1^ h^–1^. In contrast to results that were previously obtained with *K. marxianus* and *S. cerevisiae* (Dekker *et al*. 2021), a further reduction of the oxygen content of the inlet gas to below 0.5 ppm caused wash-out of the *O. parapolymorpha* chemostat cultures.

*Saccharomyces cerevisiae* can grow anaerobically in synthetic media supplemented with sterols, a UFA source and a standard vitamin solution also used for aerobic cultivation (Andreasen and Stier 1953, 1954, Dekker *et al*. 2019). Based on UFA and sterol contents of aerobically grown *S. cerevisiae* biomass, the minimum oxygen-uptake rate required for synthesis of these lipids at a specific growth rate of 0.10 h^–1^ were estimated at 0.01 mmol O_2_ (g biomass)^–1^ h^–1^ (Dekker *et al*. 2019). The biomass-specific oxygen-consumption rate of 0.60 mmol O_2_ (g biomass)^–1^ h^–1^ observed in oxygen-limited cultures of *O. parapolymorpha* (Table 3) was 60-fold higher than this estimate. Based on the assumption that oxygen-limited cultures predominantly used oxygen for respiration, oxygen-uptake and ethanol-production rates indicated that approximately 3% of the glucose consumed by these cultures was respired. Under the same oxygen-limitation regime, *S. cerevisiae* and *K. marxianus* showed specific oxygen-consumption rates below 0.25 mmol (g biomass)^–1^ h^–1^, RQ values above 50 and very low residual glucose concentrations (Table 3; Dekker *et al*. 2021).

Oxygen-limited cultures of *O. parapolymorpha* showed an over 10-fold lower biomass-specific rate of glycerol production than similar cultures of *K. marxianus* and *S. cerevisiae* (0.02 versus 1.12 and 0.45 mmol g (biomass)^–1^ h^–1^, respectively, Table 3). In multiple yeast species, glycerol production plays a key role during anaerobic and oxygen-limited growth by enabling reoxidation of surplus NADH formed in biosynthetic reactions (Scheffers 1966, van Dijken and Scheffers 1986, Weusthuis *et al*. 1994, Bakker *et al*. 2001), which, under aerobic conditions, is achieved by mitochondrial respiration (Bakker *et al*. 2001). In fully anaerobic cultures of *S. cerevisiae* and in severely oxygen-limited *K. marxianus* cultures, 7–12 mmol glycerol was formed per gram of yeast biomass (Dekker *et al*. 2021). At D = 0.1 h^–1^, reoxidation of an equivalent amount of NADH by respiration would require an uptake rate of 0.4–0.6 mmol O_2_ g (biomass)^–1^ h^–1^, which corresponds well with the observed oxygen consumption rates of the oxygen-limited *O. parapolymorpha* chemostat cultures (Table 3).

In some yeasts, an insufficient capacity for glycerol production has been linked to an inability to grow under severe oxygen limitation. Scheffers (1963, 1966) showed that this phenomenon, which he labeled the Custers effect, no longer occurred when cultures were supplemented with acetoin. Similarly, in *S. cerevisiae*, NADH-dependent reduction of acetoin by the 2,3-butanediol dehydrogenase Bdh1 (Gonzalez *et al*. 2000) restores fermentation of glycerol-negative strains (Björkqvist *et al*. 1997). Presence of an ScBdh1 ortholog in the predicted proteome of *O. parapolymorpha* CBS11895 (HPODL_00988; Figure S1, Supporting Information) indicated that this enzyme activity also occurs in *O. parapolymorpha*.

Addition of acetoin to oxygen-limited chemostat cultures of *O. parapolymorpha* led to an increase of the steady-state biomass concentration from 0.62 to 1.57 g l^–1^. A higher rate of ethanol production, a higher biomass yield on oxygen and a higher RQ (Table 4) indicated that acetoin addition led to a more fermentative metabolism. Although the biomass-specific ethanol production rates in acetoin-supplemented cultures (5.9 mmol g_x_^–1^ h^–1^, Table 4) approached those of oxygen-limited cultures of *S. cerevisiae* CEN.PK113-7D grown at the same dilution rate (7.5 mmol g_x_^–1^ h^–1^, Table 3), almost half of the glucose in the cultures remained unused. In addition, biomass-specific rates of acetoin consumption (0.97 mmol g_x_^–1^ h^–1^) in the *O. parapolymorpha* cultures were much higher than the rates estimated to be required for reoxidation of NADH generated in biosynthetic reactions. This observation suggested that 2,3-butanediol dehydrogenase activity in *O. parapolymorpha* not only reoxidized NADH formed in biosynthetic reactions but also NADH derived from sugar dissimilation. As a consequence, it would compete for NADH with alcohol dehydrogenase. Furthermore, we cannot exclude that, in these cultures, 2,3-butanediol dehydrogenase also used NADPH generated in the oxidative pentose-phosphate pathway or other NADP^+^-dependent oxidative processes or reactions. This notwithstanding, these results clearly implicated a limited capacity for NADH reoxidation as a key factor in the unexpectedly large oxygen requirements of *O. parapolymorpha*.

**Table 4.** Physiological parameters of glucose-grown, oxygen-limited chemostat cultures (D = 0.1 h^–1^, 30°C) of *O. parapolymorpha* strains expressing the *S. cerevisiae* glycerol pathway genes Sc*GPP1* and/or Sc*GPD2* or supplemented with 2.0 g l^–1^ acetoin. Data are represented as mean ± standard deviation of data obtained from replicate chemostat cultures. Negative and positive biomass-specific conversion rates (q) represent consumption and production rates, respectively, with subscript x denoting biomass dry weight. B.D.: below detection limit (concentration < 0.1 mM) and (-): not applicable due to co-consumption of ethanol added as ergosterol solvent.

Yeast strain	CBS11895	IMX2119	IMX2587	IMX2588	CBS11895
Relevant genotype	Wild type	*ScGPP1*	*ScGPD2*	*ScGPP1*	
*ScGPD2*	Wild type				
O_2_ in inlet gas (ppm)	840	840	840	840	840
Acetoin in feed (mM)	0	0	0	0	23
Replicates	2	3	2	3	3
Biomass (g_x_ l^–1^)	0.62 ± 0.01	0.91 ± 0.06	0.66 ± 0.09	0.86 ± 0.09	1.57 ± 0.08
Residual glucose (g l^–1^)	15.92 ± 0.01	14.33 ± 0.61	15.71 ± 0.57	14.98 ± 0.64	9.41 ± 0.41
**Biomass-specific conversion rates**					
Specific growth rate (h^–1^)	0.10 ± 0.00	0.11 ± 0.01	0.10 ± 0.01	0.12 ± 0.01	0.11 ± 0.00
q_glucose_ (mmol g_x_^–1^ h^–1^)	−3.67 ± 0.20	−3.69 ± 0.43	−2.97 ± 0.18	−4.16 ± 0.19	−4.11 ± 0.12
q_ethanol_ (mmol g_x_^–1^ h^–1^)	4.75 ± 0.33	4.92 ± 0.52	4.72 ± 0.20	7.26 ± 0.57	5.90 ± 0.40
q_glycerol_ (mmol g_x_^–1^ h^–1^)	0.02 ± 0.01	0.18 ± 0.02	0.03 ± 0.01	0.22 ± 0.01	0.03 ± 0.00
q_succinate_ (mmol g_x_^–1^ h^–1^)	B.D.	B.D.	0.02 ± 0.02	0.01 ± 0.02	0.02 ± 0.00
q_acetoin_ (mmol g_x_^–1^ h^–1^)	-	-	-	-	−0.97 ± 0.04
q_butanediol_ (mmol g_x_^–1^ h^–1^)	-	-	-	-	0.97 ± 0.06
q_O2_ (mmol g_x_^–1^ h^–1^)	−0.60 ± 0.01	−0.44 ± 0.03	−0.59 ± 0.06	−0.37 ± 0.04	−0.29 ± 0.01
q_CO2_ (mmol g_x_^–1^ h^–1^)	6.42 ± 0.30	6.41 ± 0.80	5.33 ± 0.37	6.45 ± 0.42	7.00 ± 0.41
**Stoichiometries**					
RQ (-qCO_2_/qO_2_)	10.7 ± 0.3	14.4 ± 0.9	9.11 ± 0.23	17.4 ± 1.6	24.2 ± 0.9
Y_glycerol/X_ (mmol g_x_^–1^)	0.24 ± 0.07	1.71 ± 0.09	0.30 ± 0.12	1.93 ± 0.25	0.32 ± 0.04
Y_X/glucose_ (g_x_ g^–1^)	0.15 ± 0.02	0.16 ± 0.00	0.19 ± 0.01	0.16 ± 0.01	0.14 ± 0.00
Y_ethanol/glucose_ (g g^–1^)	0.33 ± 0.04	0.34 ± 0.01	0.41 ± 0.00	0.45 ± 0.01	0.37 ± 0.02
Y_X/O2_ (g_x_ mmol^–1^)	0.17 ± 0.01	0.24 ± 0.01	0.18 ± 0.03	0.32 ± 0.05	0.37 ± 0.00
**Recoveries (out/in)**					
Carbon (%)	94.4 ± 6.2	98.4 ± 2.2	102.8 ± 1.7	102.4 ± 2.8	96.2 ± 1.3
Degree of reduction (%)	92.0 ± 6.1	93.5 ± 3.1	102.9 ± 0.4	106.0 ± 3.1	95.6 ± 1.1

### Absence of orthologs of *S. cerevisiae* glycerol-3P phosphatase in Ogataea species

**Figure 1.**
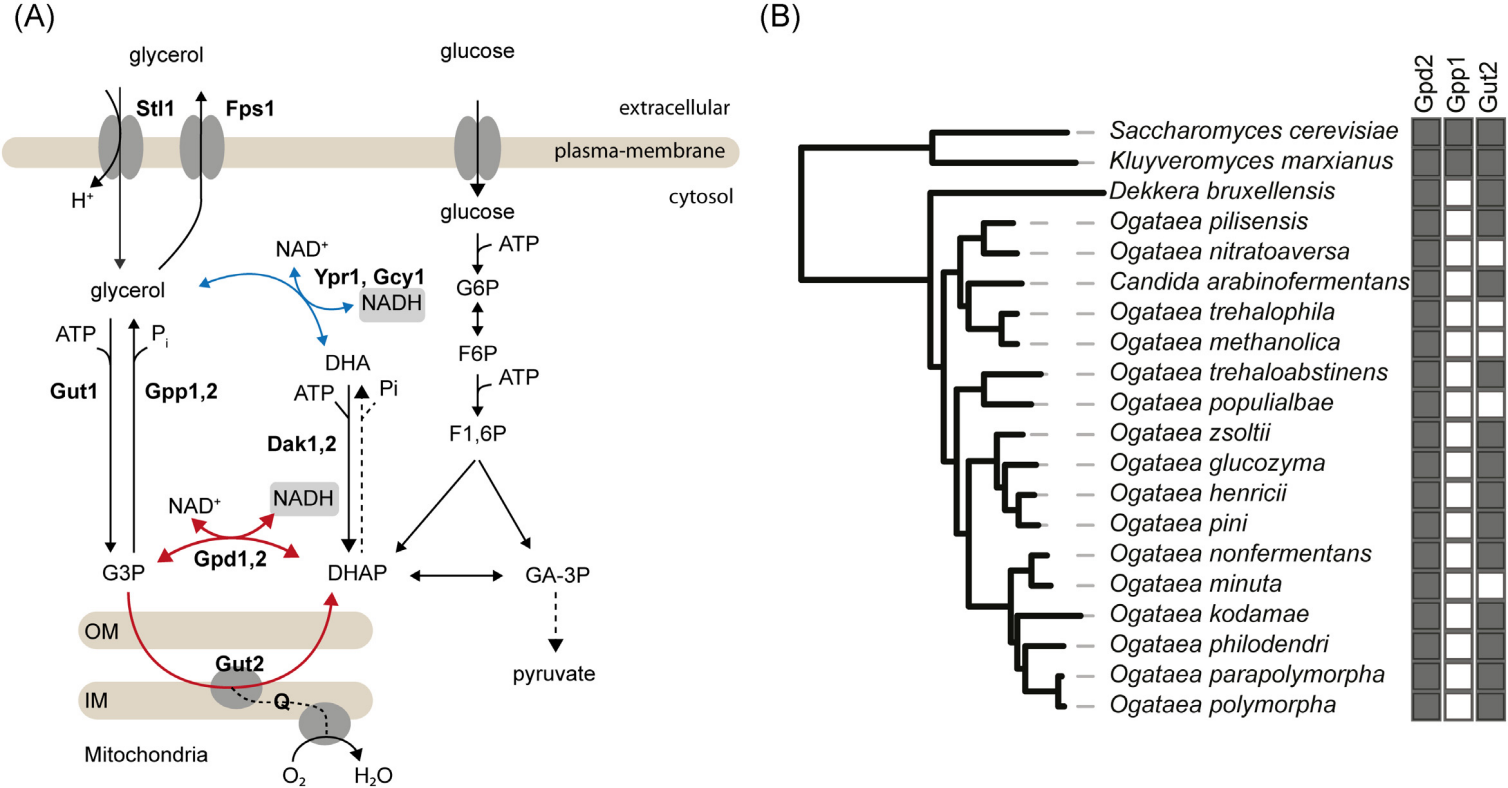
**(A)** Reactions and proteins involved in glycerol metabolism in *S. cerevisiae*. Gpd1 is mainly located in peroxisomes and Gpd2 in the cytosol and in mitochondria (Valadi *et al*. 2004). Red arrows represent the glycerol-3-phosphate shuttle, the dashed arrow linking DHAP and DHA indicates the hypothetical formation, in non-*Saccharomyces* yeasts, of glycerol via DHAP phosphatase and NAD(P)H-dependent DHA reductase (blue arrow; Klein *et al*. 2017). **(B)** Occurrence of orthologs of *S. cerevisiae* structural genes encoding glycerol-3P dehydrogenase (Gpd2), glycerol-3P phosphatase (Gpp1), and FAD-dependent mitochondrial glycerol-3P dehydrogenase (Gut2) in *Ogataea* sp., *Brettanomyces* (syn. *Dekkera*) *bruxellensis, K. marxianus*, and *S. cerevisiae*. Black and white squares indicate presence and absence, respectively, of orthologs, based on homology searches of whole-genome translated sequences with *S. cerevisiae* S288c sequences as queries. Species are mapped to the phylogenetic tree of *Saccharomycotina* yeasts (Shen *et al*. 2020).

To study the molecular basis for the near absence of glycerol formation in oxygen-limited cultures of *O. parapolymorpha*, we investigated presence of orthologs of *S. cerevisiae GPD1/2* and *GPP1/2* in genomes of *Ogataea* species. These genes encode isoenzymes that catalyze the two key reactions of the *S. cerevisiae* glycerol pathway, NAD^+^-dependent glycerol-3P dehydrogenase and glycerol-3P phosphatase, respectively (Albertyn *et al*. 1992, Norbeck *et al*. 1996, Ansell *et al*. 1997). A homology search in translated whole-genome sequences of 16 *Ogataea* species (Shen *et al*. 2020) revealed clear Gpd orthologs, but no Gpp orthologs (Fig. 1). In this respect, *Ogataea* yeasts resembled the phylogenetically related genus *Brettanomyces* (syn. *Dekkera*; Fig. 1), whose representatives are known to exhibit a Custers effect (Wijsman *et al*. 1984, Galafassi *et al*. 2013). In the absence of glycerol-3P phosphatase, NAD^+^-dependent glycerol-3P dehydrogenase can still contribute to glycerolipid synthesis (Athenstaedt *et al*. 1999) and participate in the glycerol-3P shuttle for coupling oxidation of cytosolic NADH to mitochondrial respiration (Larsson *et al*. 1998, Overkamp *et al*. 2000, Rigoulet *et al*. 2004; Fig. 1).

### Transcriptional responses of *O. parapolymorpha* to oxygen limitation

Responses of *O. parapolymorpha* to oxygen limitation were further explored by transcriptome analyses on aerobic and oxygen-limited chemostat cultures. The resulting transcriptome data were first used to refine the genome annotation of a *de novo* assembled genome sequence of *O. parapolymorpha* CBS11895 obtained from long-read sequence data (see Data availability).

**Figure 2.**
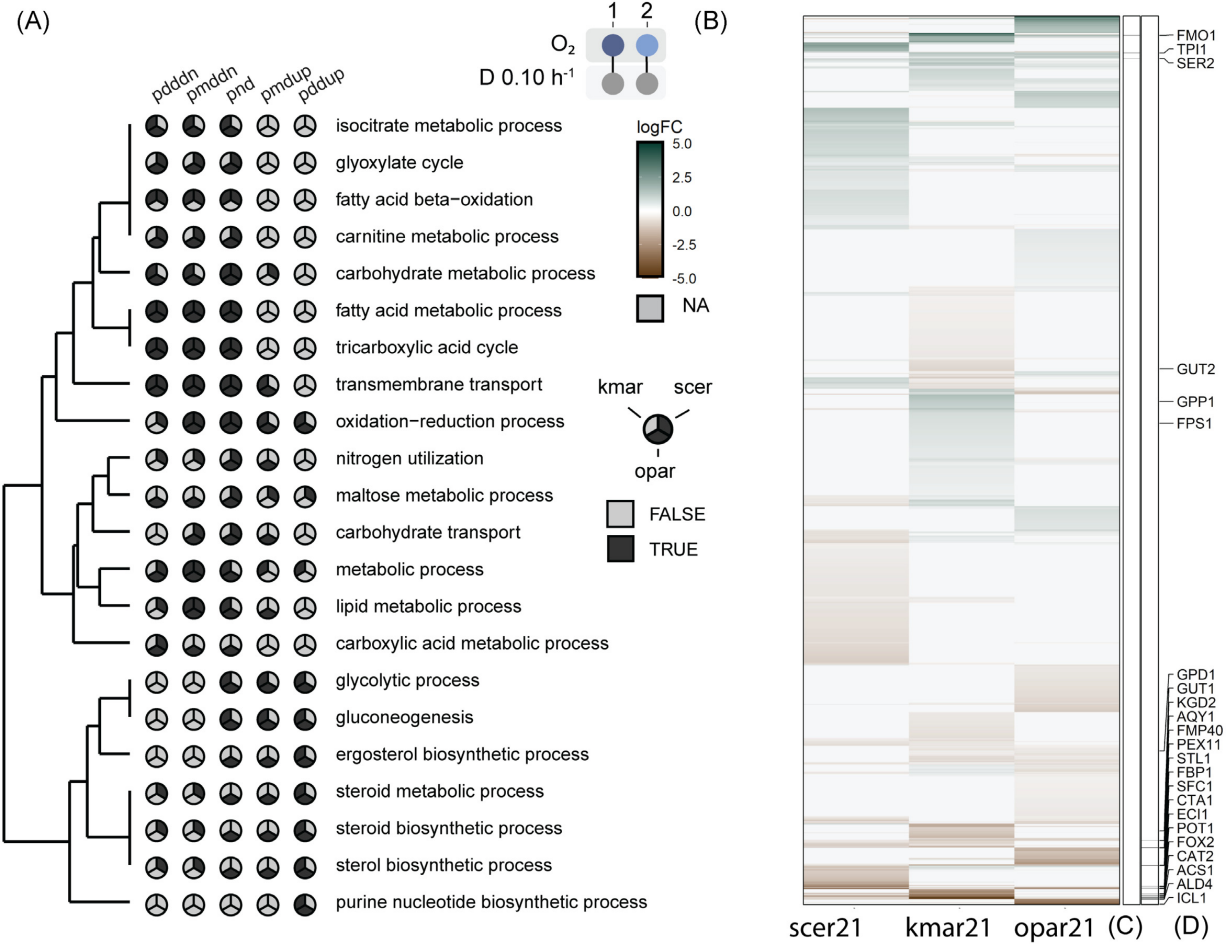
Genome-wide transcriptional responses of *O. parapolymorpha* (opar), *K. marxianus* (kmar), and *S. cerevisiae* (scer) to oxygen limitation. Aerobic (regime 1, 21 × 10^4^ ppm O_2_ in inlet gas, and 7.5 g l^–1^ glucose in feed medium) and oxygen-limited (regime 2, 840 ppm O_2_ in inlet gas; 20 g l^–1^ glucose in feed medium) chemostat cultures were grown at D = 0.1 h^–1^ and 30°C. Data for *K. marxianus* and *S. cerevisiae* were obtained from a previous study (Dekker *et al*. 2021). **(A)** Gene-set enrichment analysis showing GO-terms overrepresented among genes showing a transcriptional response to oxygen limitation (regime 2 versus regime 1) in at least two of the three yeast species. Distinct directionalities calculated with Piano (Väremo *et al*. 2013) are indicated as distinct-directional down (pdddn), mixed-directional down (pmddn), nondirectional (pnd), mixed-directional up (pmdup), and distinct-directional up (pddup). Hierarchical clustering was based on degree of overrepresentation. Data on all enriched GO-terms for biological processes are shown in Figures S2–S4 (Supporting Information). **(B)** Log-fold changes (regime 2 versus regime 1) of orthologs in the three yeasts, **(C)** Orthologs showing higher transcript levels in oxygen-limited cultures of all three yeasts. **(D)** Orthologs showing lower transcript levels in oxygen-limited cultures of all three yeasts.

Transcriptional responses of *O. parapolymorpha* to oxygen limitation were compared to those of *S. cerevisiae* and *K. marxianus* (Dekker *et al*. 2021) grown under the same aeration regimes. A global comparison at the level of functional categories indicated large differences in the transcriptional responses of these three yeasts to oxygen limitation (Fig. 2A). Of genes for which orthologs occur in all three species (Fig. 2B), only very few showed a consistent cross-species transcriptional response to oxygen limitation (Fig. 2C and D). At first glance, these different transcriptional responses suggested a completely different wiring of their oxygen-responsive transcriptional regulation networks. Based on functional categories, the only shared global transcriptional responses of *O. parapolymorpha, K. marxianus*, and *S. cerevisiae* were a downregulation, in the oxygen-limited cultures, of genes involved in the metabolism of nonglucose carbon sources (GO categories fatty-acid metabolic process, tricarboxylic acid cycle, transmembrane transport, metabolic process, and lipid metabolic process; Fig. 2A). These responses are in line with the requirement for oxygen in the dissimilation of nonfermentable substrates and for a key role of the tricarboxylic acid cycle in respiratory glucose metabolism. However, in addition to oxygen availability, different glucose and ethanol concentrations in chemostat cultures of the tested yeast strains (Table 3) may have had a strong impact on transcript profiles. For example, in comparisons of glucose-limited and glucose-sufficient chemostat cultivation regimes, hundreds of *S. cerevisiae* genes were shown to exhibit an at least 2-fold difference in transcript level (Meijer *et al*. 1998, Boer *et al*. 2003, Tai *et al*. 2005). In view of this intrinsic limitation of the chemostat-based transcriptome studies, analysis was focused on genes and pathways that were previously implicated in biosynthetic oxygen requirements of yeasts.

Sterol biosynthesis requires molecular oxygen and, under anaerobic conditions, *S. cerevisiae* can acquire ergosterol from the media. In contrast to *S. cerevisiae*, which showed downregulation of genes associated with sterol metabolism in oxygen-limited cultures, GO-term enrichment analysis showed upregulation of genes associated with this process in *O. parapolymorpha* and *K. marxianus* (Fig. 2A). *Kluyveromyces marxianus* and several other pre-WGD yeast species lack a functional sterol-import system (Dekker *et al*. 2021, Tesnière *et al*. 2021). Upregulation of sterol synthesis genes in oxygen-limited, sterol-supplemented cultures (Fig. 2A), as well as absence of clear orthologs of the *S. cerevisiae AUS1* and *PDR11* sterol-importer genes in its genome, suggested that the same holds for *O. parapolymorpha*.

**Figure 3.**
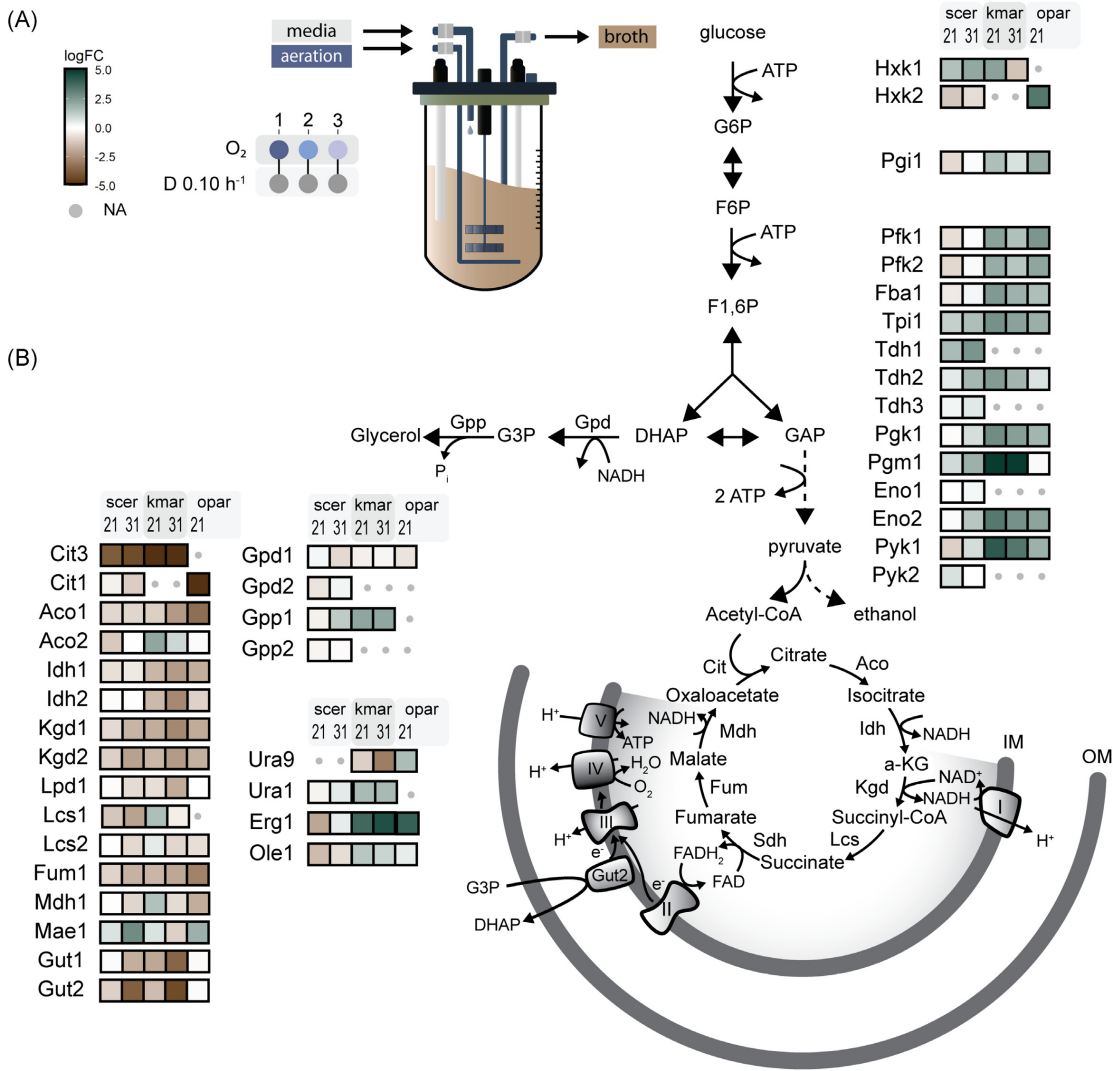
Transcriptional regulation of specific pathways and genes in *O. parapolymorpha, K. marxianus*, and *S. cerevisiae* subjected to different aeration regimes. **(A)** Chemostat cultures were grown on glucose at D = 0.1 h^–1^ and 30°C. Regime 1 (aerobic): 21 × 10^4^ ppm O_2_ in inlet gas, 7.5 g l^–1^ glucose in feed medium); Regime 2 (oxygen limitation): 840 ppm O_2_ in inlet gas; 20 g l^–1^ glucose in feed; and Regime 3 (extreme oxygen limitation): < 0.5 ppm O_2_ medium 20 g l^–1^ glucose in feed). *Ogataea parapolymorpha* washed out under regime 3. Data for *K. marxianus* and *S. cerevisiae* were obtained from a previous study (Dekker *et al*. 2021). In comparisons of transcript levels Regime 1 was used as the reference. **(B)** Single biochemical reactions are represented by arrows, lumped reactions by dashed arrows; some metabolites and cofactors are omitted to facilitate visualization. Respiratory complexes are indicated by Roman numerals. The *O. parapolymorpha* genome encodes all subunits of Complex I (Riley *et al*. 2016), which is absent in *S. cerevisiae*. Colored boxes indicate upregulation (blue-green) or downregulation (brown), color intensities indicate log 2 fold-change (log FC, capped to a maximum value of 5). Enzymes are indicated as *S. cerevisiae* orthologs; absence of orthologs in the other yeasts is indicated by grey dots. Abbreviations: G6P, glucose-6-phosphate; F6P, fructose-6-phosphate; F1,6P, fructose-1,6-bisphosphate; DHAP, dihydroxyacetone phosphate; G3P, glycerol-3-phosphate; GAP, glyceraldehyde-3-phosphate; IM, inner mitochondrial membrane; and OM, outer mitochondrial membrane.

A recent study confirmed that Op*URA9*, which encodes the respiratory-chain-linked Class-II DHODase of *O. parapolymorpha*, complements the uracil auxotrophy of *ura1Δ S. cerevisiae* under aerobic, but not under anaerobic conditions (Bouwknegt *et al*. 2021). Op*URA9* showed higher transcript levels in oxygen-limited cultures than in aerobic cultures, while its *K. marxianus* ortholog Km*URA9* showed the reverse response (Fig. 3). This observation is consistent with the presence and absence of a respiration-independent Class-I-A DHODase in *K. marxianus* and *O. parapolymorpha*, respectively (Bouwknegt *et al*. 2021, Dekker *et al*. 2021).

The importance of glycerol production in oxygen-limited cultures of *S. cerevisiae* and *K. marxianus* was evident from an upregulation of *GPP1* (Fig. 3), for which no ortholog was found in *O. parapolymorpha* (Fig. 1). Lack of a transcriptional response of the single *GPD* ortholog to oxygen limitation are consistent and further supports the notion that *O. parapolymorpha* does not use glycerol formation as a redox sink during oxygen-limited growth.

### Glycerol production in aerobic cultures of an *O. parapolymorpha* strain lacking mitochondrial glycerol-3P dehydrogenase and alternative NADH dehydrogenases

Presence of orthologs of *S. cerevisiae GPD1/2* and *GUT2* in the *O. parapolymorpha* genome suggested possible involvement of a glycerol–phosphate shuttle (Larsson *et al*. 1998) in respiratory oxidation of cytosolic NADH. To investigate whether elimination of systems for mitochondrial, respiratory oxidation of NADH would affect glycerol production by *O. parapolymorpha*, we studied growth and product formation in strain IMX2167. In this strain, Op*GUT2* and the genes encoding three cytosol- and matrix-facing alternative mitochondrial NADH dehydrogenases were deleted, while leaving the Complex-I NADH dehydrogenase complex intact (Juergens *et al*. 2021). In aerobic chemostat cultures grown at D = 0.1 h^–1^, conversion rates of strain IMX2167 were not substantially different from those of the wild-type strain CBS11895 (Table 5). Apparently, as observed in aerobic cultures of corresponding mutant strains of *S. cerevisiae* (Bakker *et al*. 2001), an ethanol–acetaldehyde shuttle and/or other redox-shuttle systems for mitochondrial oxidation of cytosolic NADH compensated for absence of a Gpp ortholog and external NADH dehydrogenases in strain IMX2167. In this strain, which also lacked the internal alternative NADH dehydrogenase, such a shuttle mechanism could couple oxidation of cytosolic NADH to the Complex-I NADH dehydrogenase (Bakker *et al*. 2001).

**Table 5.** Physiological parameters of glucose-grown aerobic bioreactor-batch and chemostat cultures (30°C) of wild-type *O. parapolymorpha* and strains carrying null mutations in genes involved in mitochondrial oxidation of NADH. Batch culture data were derived from analyses on samples taken during the exponential growth phase. Data on aerobic chemostat cultures were derived from a separate study (Juergens *et al*. 2021). Chemostat cultures (D = 0.1 h^–1^) and batch cultures were grown on 20 g l^–1^ glucose and 7.5 g l^–1^ glucose, respectively. Data are represented as mean ± standard deviation of data obtained from replicate cultures. Negative and positive biomass-specific conversion rates (q) represent consumption and production rates, respectively, with subscript x denoting biomass dry weight. B.D.: below detection limit (concentration < 0.1 mM) and N.D.: not determined.

*O. parapolymorpha* strain	CBS11895	IMX2167	CBS11895	IMX2167
Relevant genotype	Wld type	*ndh1-3*Δ *gut2*Δ	Wild type	*ndh1-3*Δ *gut2*Δ
Replicates	2	2	2	2
Cultivation mode	Batch	Batch	Chemostat	Chemostat
**Biomass-specific conversion rates**				
Specific growth rate (h^–1^)	0.36 ± 0.01	0.26 ± 0.00	0.10 ± 0.00	0.10 ± 0.00
q_glucose_ (mmol g_x_ h^–1^)	−3.90 ± 0.21	−2.73 ± 0.08	−1.08 ± 0.04	−1.04 ± 0.00
q_ethanol_ (mmol g_x_ h^–1^)	B.D.	0.22 ± 0.11	B.D.	B.D.
q_glycerol_ (mmol g_x_ h^–1^)	B.D.	0.34 ± 0.01	B.D.	B.D.
q_O2_ (mmol g_x_ h^–1^)	N.D.	−3.65 ± 0.07	−2.69 ± 0.07	−2.14 ± 0.00
q_CO2_ (mmol g_x_ h^–1^)	N.D.	4.34 ± 0.02	2.82 ± 0.04	2.25 ± 0.00
**Stoichiometries**				
RQ (-qCO_2_/qO_2_)	N.D.	1.19 ± 0.03	1.05 ± 0.01	1.05 ± 0.00
Y_X/glucose_ (g_x_ g^–1^)	0.52 ± 0.01	0.51 ± 0.01	0.51 ± 0.00	0.52 ± 0.00
Y_X/O2_ (g_x_ mmol^–1^)	N.D.	0.07 ± 0.00	0.04 ± 0.00	0.05 ± 0.00
**Recoveries (out/in)**				
Carbon (%)	N.D.	97.8 ± 1.4	99.3 ± 1.7	98.7 ± 0.4

In aerobic batch cultures, strain IMX2167 grew slower than the wild-type strain CBS11895 (0.26 h^–1^ and 0.36 h^–1^, respectively, Table 5). Glycerol production by strain IMX2167 suggested that, while lacking an Sc*GPP1* ortholog, *O. parapolymorpha* contains an alternative glycerol-3-phosphatase. Annotation of the newly assembled genome sequence of strain CBS11895 yield 24 genes annotated with the GO-term ‘phosphatase activity’ (GO:0016791). While all 24 were transcribed (log Counts per million (CPM) > 3.5), none showed significantly higher (log FC > 2) transcript levels in strain IMX2167 than in the wild-type strain CBS11895 (Table S2 and Figures S5 and S6, Supporting Information).

### Engineering of glycerol metabolism in *O. parapolymorpha*

**Figure 4.**
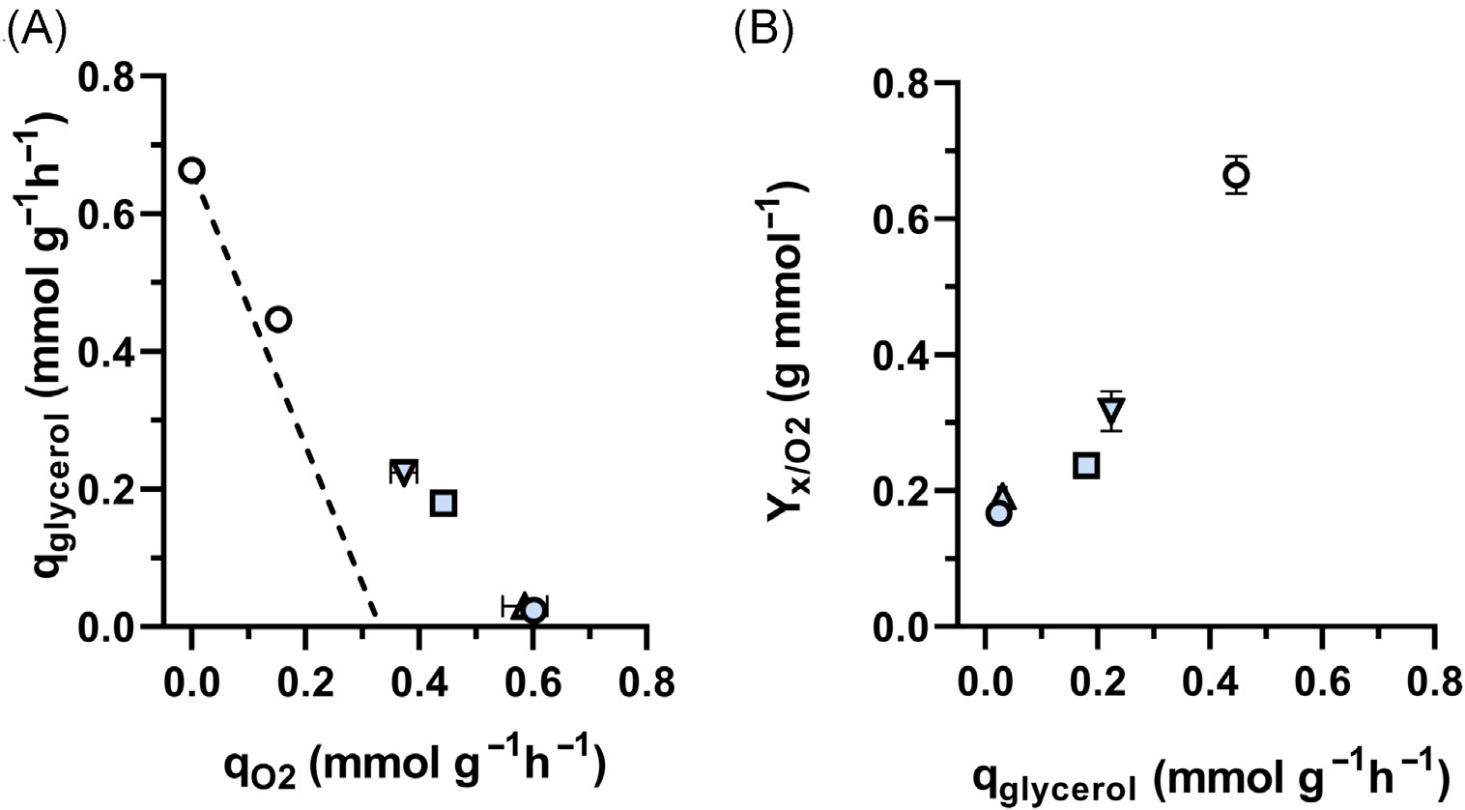
Impact of expression of Sc*GPP1* and/or Sc*GPD2* in *O. parapolymorpha* CBS11895 on glycerol production in oxygen-limited chemostat cultures. Biomass-specific conversion rates (q) were measured in oxygen-limited chemostat cultures of *O. parapolymorpha* strains (D = 0.1 h^–1^; 840 ppm O_2_ in inlet gas; and 20 g l^–1^ glucose in feed medium). Data on anaerobic and oxygen-limited chemostat cultures of *S. cerevisiae* CEN.PK113-7D were derived from (Dekker *et al*. 2021). Symbols: white circles, *S. cerevisiae* CEN.PK113-7D; blue circles, *O. parapolymorpha* CBS11895; blue boxes IMX2119 *O. parapolymorpha* (Sc*GPP1*), blue triangles up *O. parapolymorpha* IMX2587 (Sc*GPD2*), and blue triangles down *O. parapolymorpha* IMX2588 (Sc*GPP1* Sc*GPD2*). Data are represented as mean ± standard deviation of data obtained from independent chemostat cultures of each strain. (**A**) Biomass-specific glycerol production rates versus biomass-specific oxygen consumption rates. The dashed line depicts a stoichiometric relationship between glycerol production and oxygen consumption in *S. cerevisiae* cultures, based on the assumption that, for NADH reoxidation, consumption of one mol O_2_ corresponds to production of 2 moles of glycerol (Weusthuis et al. 1994). (**B**) Biomass yields on oxygen versus biomass-specific rates of glycerol production.

Based on the absence of orthologs of *S. cerevisiae* Sc*GPP1* in genomes of *Ogataea* sp. (Fig. 1B), we investigated whether expression of *ScGPP1* in *O. parapolymorpha* supported glycerol production by oxygen-limited cultures. An expression cassette in which the coding region of Sc*GPP1* was expressed from the Op*PMA1* promoter (Juergens *et al*. 2020) was integrated into the genome of *O. parapolymorpha* CBS11895. The resulting strain IMX2119 showed a 9-fold higher biomass-specific rate of glycerol formation in oxygen-limited cultures than the wild-type strain (0.18 mmol (g biomass)^–1^ h^–1^ and 0.02 mmol (g biomass)^–1^ h^–1^, respectively, Table 4 and Fig. 4A). A further increase of the glycerol production rate to 0.22 mmol (g biomass)^–1^ h^–1^ was observed when Sc*GPP1* expression was combined with integration of a cassette in which Sc*GPD2* was expressed from the Op*TEF1* promoter (Juergens *et al*. 2020; strain IMX2588; Table 4 and Fig. 4A). Integration of only the Sc*GPD2* cassette (strain IMX2587) did not result in a significantly higher rate of glycerol production in oxygen-limited cultures than observed for the wild-type strain (Table 4).

The higher biomass-specific rates of glycerol production by the Sc*GPP1* and Sc*GPP1*/Sc*GPD2* expressing *O. parapolymorpha* strains coincided with higher biomass yields on oxygen under oxygen-limited conditions (0.24 and 0.32 g biomass mmol O_2_^–1^, respectively, versus 0.17 g biomass mmol O_2_^–1^ for the wild-type strain; Table 4 and Fig. 4B). A larger contribution of alcoholic fermentation to glucose dissimilation was also concluded from the RQ values of strains IMX2119 and IMX2588 (14.4 and 17.4, respectively), which were higher than those of corresponding oxygen-limited cultures of the wild-type strain (RQ of 10.7, Table 4).

For fully anaerobic chemostat cultures of *S. cerevisiae* CEN.PK113-7D grown at D = 0.1 h^–1^, a biomass-specific rate of glycerol production of 0.67 mmol (g biomass)^–1^ h^–1^ was reported (Fig. 4A; Geertman *et al*. 2006, Dekker *et al*. 2021). An even higher rate of glycerol production (1.1 mmol (g biomass)^–1^ h^–1^) was reported for strains of another *S. cerevisiae* lineage grown under these conditions (Weusthuis *et al*. 1994, Nissen *et al*. 2000). Assuming that biomass composition and biosynthetic pathways in *S. cerevisiae* CEN.PK113-7D and *O. parapolymorpha* CBS11895 lead to a similar net generation of NADH, the glycerol production rate of the Sc*GPP1*/Sc*GPD2* expressing *O. parapolymorpha* strain IMX2588 remained approximately 4-fold lower than needed for reoxidation of all NADH generated in biosynthesis. A limiting capacity of the engineered glycerol pathway was further indicated by the residual glucose concentrations in oxygen-limited cultures of strain IMX2588, which were higher than in acetoin-supplemented cultures of the wild-type strain CBS11895 (Table 4).

## Discussion

This study revealed a surprisingly high oxygen requirement in oxygen-limited cultures of the facultatively fermentative yeast *O. parapolymorpha* (previously *Hansenula polymorpha*; Kurtzman 2011) relative to those previously reported for the pre-WGD yeasts *Kluyveromyces marxianus, K. lactis*, and *Candida utilis* (*Cyberlindnera jadinii*; Weusthuis *et al*. 1994, Kiers *et al*. 1998, Dekker *et al*. 2021). Very low glycerol-production rates and a strong impact of acetoin co-feeding to oxygen-limited cultures identified reoxidation of NADH, formed in biosynthetic reactions, as a key contributor to the large oxygen requirement of *O. parapolymorpha*. A large oxygen requirement for fermentative growth (‘Custers effect’; Wikén *et al*. 1961), absence of glycerol production and a stimulating effect of acetoin on oxygen-limited growth were previously observed in *Brettanomyces* (*Dekkera*) yeasts (Custers 1940, Wikén *et al*. 1961, Scheffers 1966, Wijsman *et al*. 1984). The Custers effect in *B. bruxellensis* was attributed to absence of glycerol-3P phosphatase activity in cell extracts (Wijsman *et al*. 1984) and lack of an ortholog of the *S. cerevisiae GPP1/GPP2* genes (Tiukova *et al*. 2013). The genera *Ogataea* and *Brettanomyces* both belong to the Pichiacaea family (Shen *et al*. 2016). Our observations on *O. parapolymorpha*, combined with the absence of clear Sc*GPP1*/Sc*GPP2* orthologs in genomes of other *Ogataea* species, provide an incentive for further studies into the occurrence, regulatory basis and ecophysiological significance of a Custers effect in Pichiacaea. In view of its fast growth in synthetic media (Juergens *et al*. 2018a) and its accessibility to genome-editing techniques (Juergens *et al*. 2018b, Gao *et al*. 2021), *O. parapolymorpha* offers an interesting experimental platform for such studies.

*Ogataea parapolymorpha* is applied in aerobic industrial processes for production of heterologous proteins (Stasyk 2017) and, based on its thermotolerance and natural ability to metabolize d-xylose, is under investigation as a potential platform organism for second-generation ethanol production (Kurylenko *et al*. 2014). In anaerobic industrial applications of *Saccharomyces* yeasts such as beer fermentation, introduction of a brief aeration phase enables yeast cell to synthesize and intracellularly accumulate sterols and UFAs, which are then used during the subsequent anaerobic fermentation phase (Casey *et al*. 1984, Meyers *et al*. 2017). The large oxygen requirements of *O. parapolymorpha* observed in this study imply that such a strategy is not feasible for this yeast. Elimination of the Custers effect in *O. parapolymorpha* is, therefore, a priority target for development of industrial ethanol-producing strains.

Formation of glycerol in aerobic cultures of strain IMX2167, in which genes encoding key enzymes of respiratory NADH oxidation, including mitochondrial glycerol-3-phosphate dehydrogenase (OpGut2), were deleted, suggested that the *O. parapolymorpha* genome may harbor a gene encoding a glycerol-3-phosphatase. Alternatively, glycerol formation in this strain may reflect activity of another pathway for glycerol production (e.g. involving DHAP phosphatase, Fig. 1). Laboratory evolution of wild-type and engineered *O. parapolymorpha* strains under oxygen-limited conditions and resequencing of evolved strains (Mans *et al*. 2018) may contribute to a better understanding of glycerol production in this yeast.

Expression of *S. cerevisiae GPP1* and *GPD2* enabled increased rates of glycerol formation and a higher biomass yield on oxygen in oxygen-limited cultures of *O. parapolymorpha* (Table 4). However, glycerol production rates were lower than observed in anaerobic cultures of *S. cerevisiae* (Fig. 4A) and a large fraction of the glucose fed to the cultures remained unused. These results indicated that the *in vivo* capacity of NADH reoxidation via heterologously expressed Gpp1 and Gpd2 was insufficient to fully replace the role of mitochondrial respiration in the reoxidation of NADH generated in biosynthetic reactions. Increased expression of *GPP1* and *GPD2*, possibly combined with expression of a glycerol exporter and/or laboratory evolution under oxygen-limited conditions can be explored to further enhance glycerol production in *O. parapolymorpha*. Alternatively, expression of heterologous pathways for NADH-dependent reduction of acetyl-CoA to ethanol (Medina *et al*. 2010) or NADH oxidation *via* a pathway involving ribulose-1,5-bisphosphatase and phosphoribulokinase (Guadalupe-Medina *et al*. 2013, Papapetridis *et al*. 2018) can be explored.

In oxygen-limited cultures of *O. parapolymorpha* that were co-fed with acetoin, incomplete glucose consumption occurred despite rates of acetoin conversion that were 2-fold higher than glycerol production rates in anaerobic *S. cerevisiae* cultures (Table 3 and Fig. 4). This result suggests that, in this yeast, not only the capacity for reoxidation of NADH generated in biosynthesis but also for NADH generated in glycolysis may be limited. This hypothesis can be tested by laboratory evolution under oxygen-limited conditions or, alternatively, by overexpression of key enzymes of pyruvate decarboxylase and/or alcohol dehydrogenase.

Predicted stoichiometric oxygen requirements for sterol synthesis and pyrimidine synthesis of *O. parapolymorpha* are small in comparison with those for NADH reoxidation. However, their physiological impacts can be augmented when key enzymes involved in these processes have a low affinity for oxygen. Absence of orthologs of the *S. cerevisiae* Aus1 and Pdr11 sterol transporters indicates that, similar to other pre-WGD yeasts (Seret *et al*. 2009), *O. parapolymorpha* is probably unable to import sterols. Due to the incompletely resolved role of cell wall proteins in sterol import in *S. cerevisiae* (Alimardani *et al*. 2004), functional expression of a heterologous system for sterol import in *O. parapolymorpha* may not be a trivial challenge. Alternatively, it may be explored whether, as shown in *S. cerevisiae* and *K. marxianus* (Wiersma *et al*. 2020, Dekker *et al*. 2021), expression of a heterologous squalene–tetrahymanol cyclase, which synthesizes the sterol surrogate tetrahymanol, can support sterol-independent growth of *O. parapolymorpha*. Genome-sequence data indicate that pyrimidine synthesis in *O. parapolymorpha* depends on a respiratory-chain-linked dihydroorate dehydrogenase (OpUra9), thus rendering pyrimidine biosynthesis in this yeast oxygen dependent (Shi and Jeffries 1998, Gojković *et al*. 2004). As previously explored in *Scheffersomyces stipitis*, expression of the soluble fumarate-coupled DHODase from *S. cerevisiae* (Ura1; Shi and Jeffries 1998) or, alternatively, of recently described respiration-independent orthologs of Ura9 (Bouwknegt *et al*. 2021) may be applied to bypass this oxygen requirement.

This study illustrates how rigorous standardization of oxygen-limited cultivation regimes (Mooiman *et al*. 2021) enables quantitative comparisons and physiological analysis of oxygen requirements of facultatively fermentative yeasts. We recently showed that enabling synthesis of a sterol surrogate sufficed to eliminate oxygen requirements of oxygen-limited *K. marxianus* cultures (Dekker *et al*. 2021). By demonstrating that oxygen requirements of *O. parapolymorpha* are much larger as well as more complex, the present study underlines the relevance of further comparative physiology studies on oxygen requirements across yeast and fungal species. Such studies are not only of fundamental scientific interest but should help to unlock the full potential of non-*Saccharomyces* yeasts for application in anaerobic industrial processes.

## Data availability

Numerical data presented in the figures in this work are available at https://figshare.com/s/283842c2a2a9a847e0bf. Raw sequencing data are available from NCBI (www.ncbi.nlm.nih.gov/geo/) under BioProject PRJNA717220.

## Code availability

Codes used to generate the results obtained in this study are archived in a Gitlab repository (https://gitlab.tudelft.nl/rortizmerino/opar_anaerobic).

## Supplementary Material

foac007_Supplemental_Figures

## Notes

Manuscript for publication in FEMS Yeast Research, special issue dedicated to the memory of W. Alexander Scheffers (1925–2021).
