# Supplementary figures and images for: Respiratory reoxidation of NADH is a key contributor to high oxygen requirements of oxygen-limited cultures of Ogataea parapolymorpha
Source: FEMS Yeast Res. 2022 Feb 7;22(1):foac007. doi: 10.1093/femsyr/foac007 (PMC8862043; doi:10.1093/femsyr/foac007)

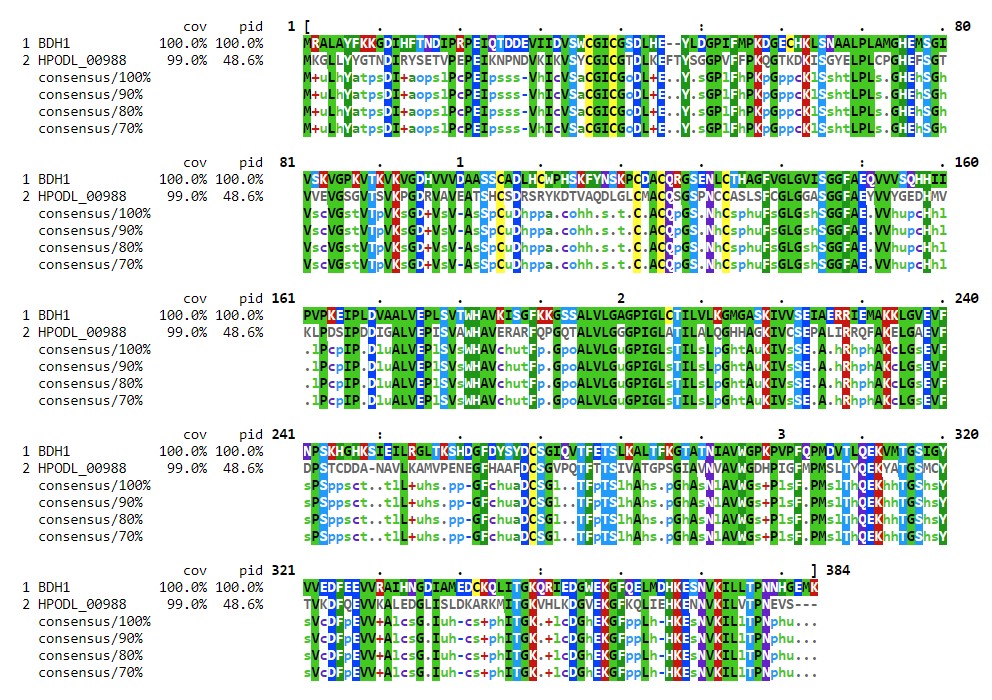

Supplement: foac007_Supplemental_Figures [file foac007_supplemental_figures.zip › fig_s1_protein_sequence_alignment_bdh1.jpeg]

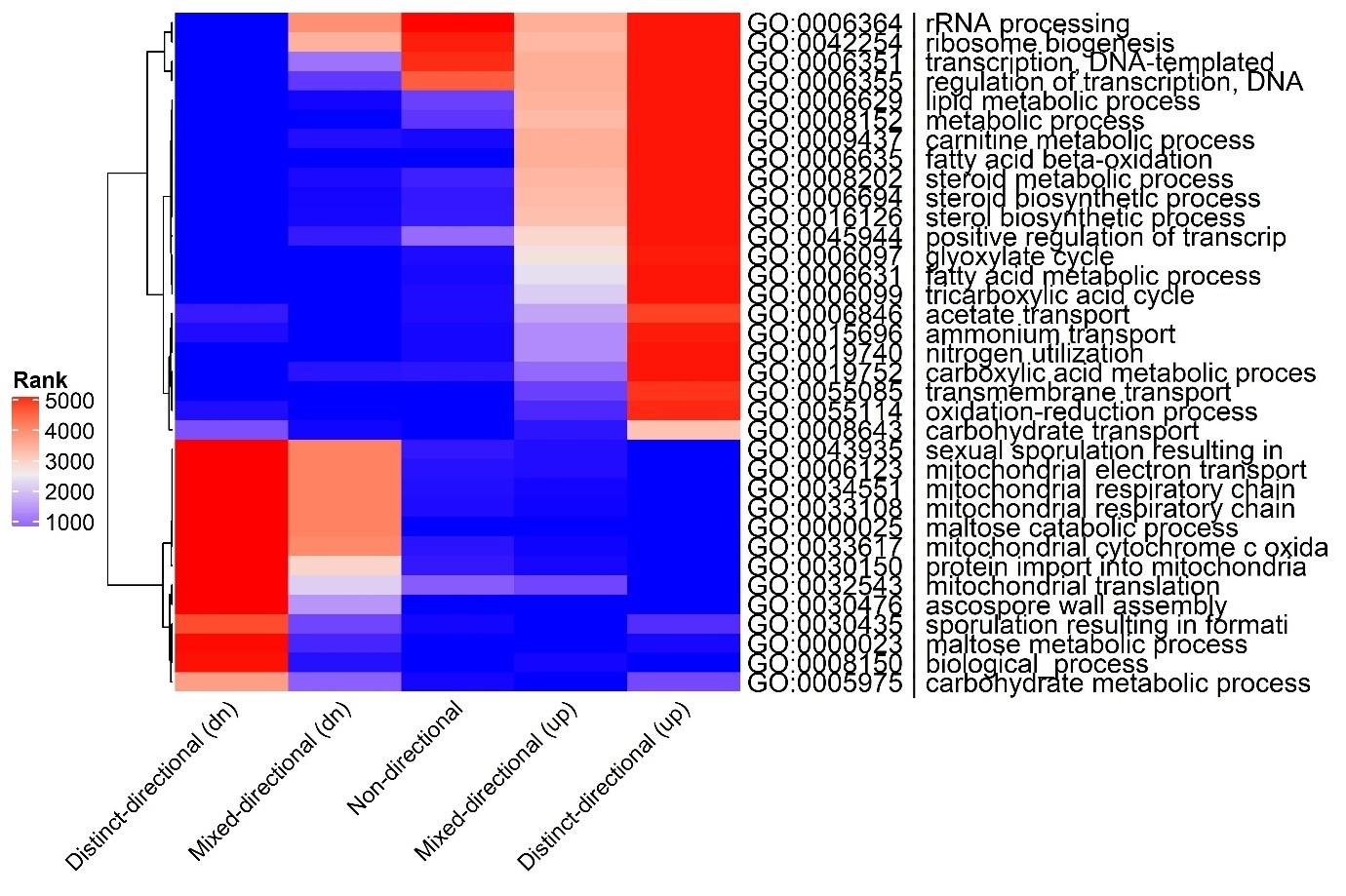

Supplement: foac007_Supplemental_Figures [file foac007_supplemental_figures.zip › fig_s2_goterm_enrichment_scere.jpeg]

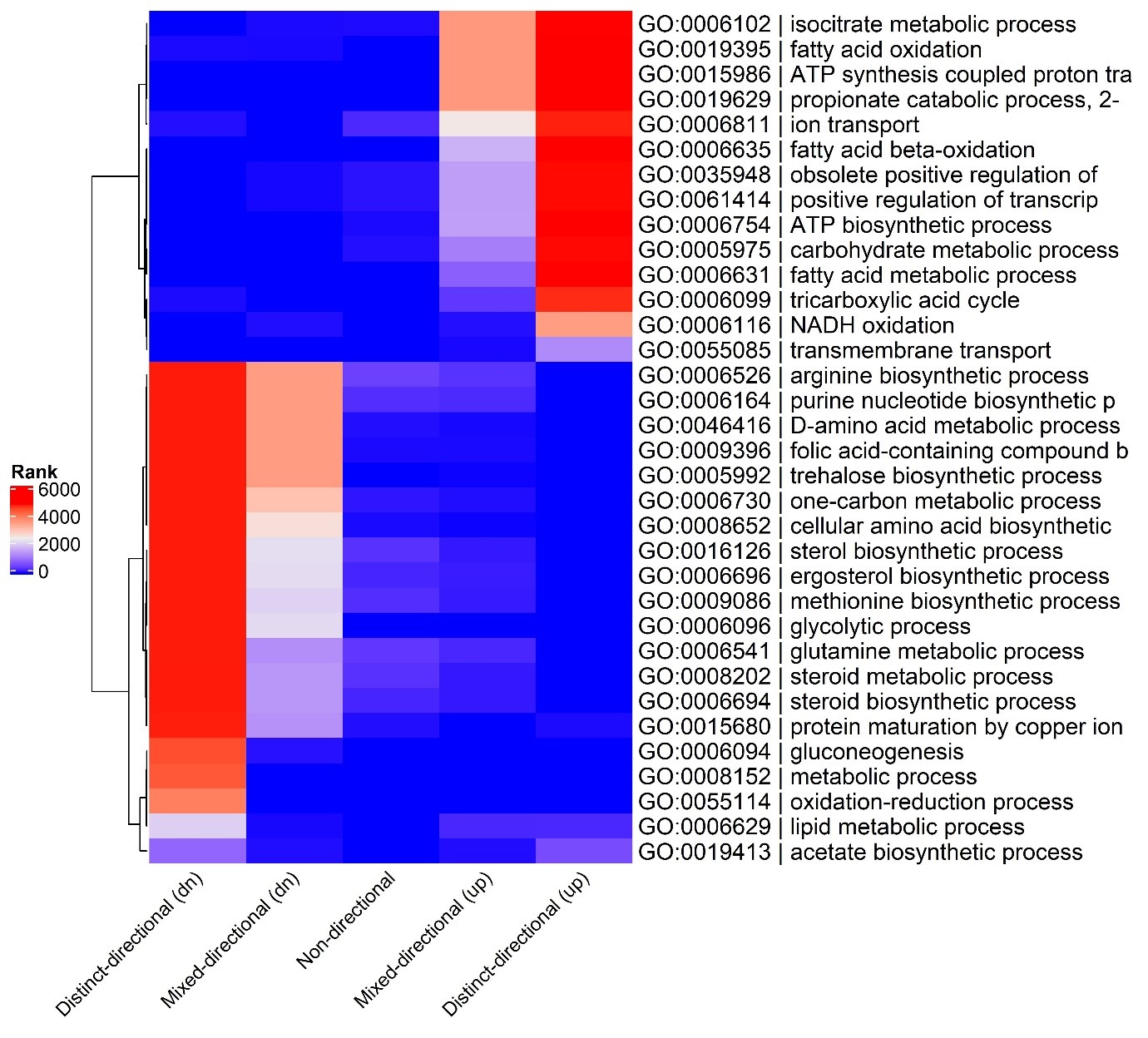

Supplement: foac007_Supplemental_Figures [file foac007_supplemental_figures.zip › fig_s3_goterm_enrichment_kmarx.jpeg]

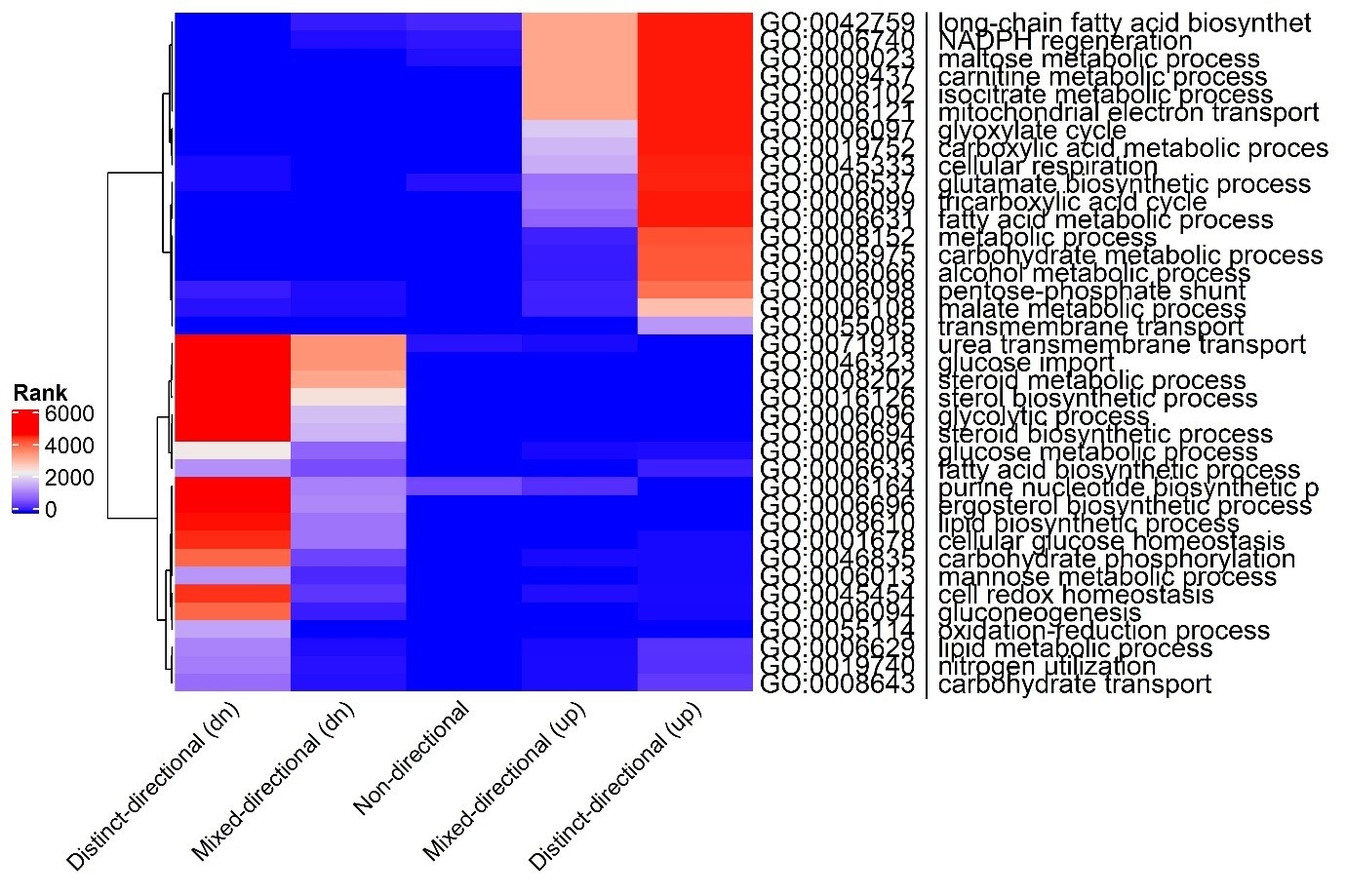

Supplement: foac007_Supplemental_Figures [file foac007_supplemental_figures.zip › fig_s4_goterm_enrichment_opara.jpeg]

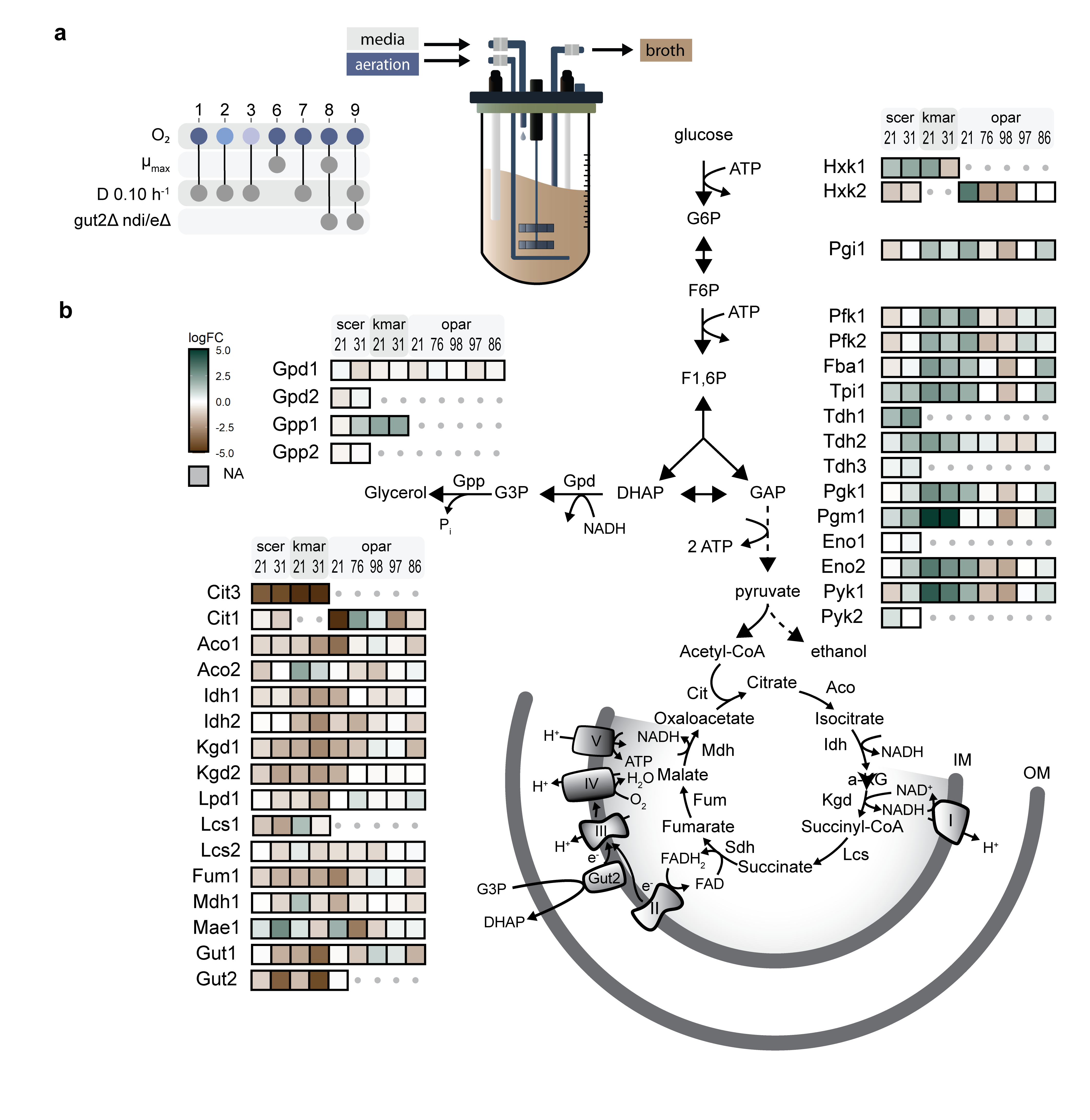

Supplement: foac007_Supplemental_Figures [file foac007_supplemental_figures.zip › fig_s5_nadh_oxidation.jpeg]

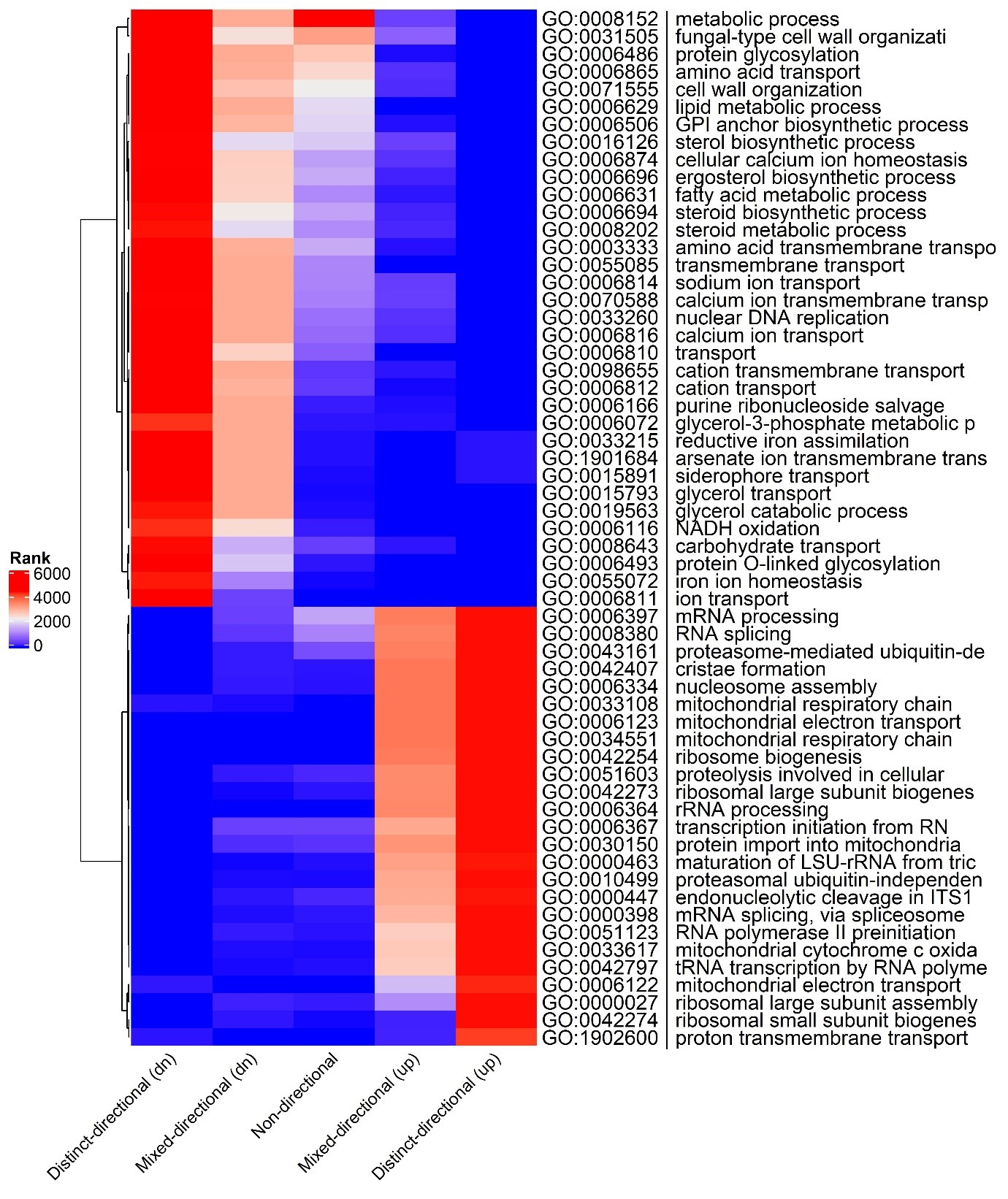

Supplement: foac007_Supplemental_Figures [file foac007_supplemental_figures.zip › fig_s6_goterm_opara_con68.jpeg]
